# A landmark‐free analysis of the pelvic girdle in Sulawesi ricefishes (Adrianichthyidae): How 2D and 3D geometric morphometrics can complement each other in the analysis of a complex structure

**DOI:** 10.1002/ece3.10613

**Published:** 2023-10-18

**Authors:** Tobias Spanke, Mariam Gabelaia, Jana M. Flury, Leon Hilgers, Letha Louisiana Wantania, Bernhard Misof, Benjamin Wipfler, Daisy Wowor, Daniel F. Mokodongan, Fabian Herder, Julia Schwarzer

**Affiliations:** ^1^ Leibniz Institute for the Analysis of Biodiversity Change (LIB) Museum Koenig Bonn Bonn Germany; ^2^ Department of Environmental Sciences University of Basel Basel Switzerland; ^3^ LOEWE‐Zentrum für Translationale Biodiversitätsgenomik Frankfurt Germany; ^4^ Faculty of Fisheries and Marine Science Sam Ratulangi University Manado Indonesia; ^5^ Museum Zoologicum Bogoriense, Research Center for Biosystematics and Evolution National Research and Innovation Agency (BRIN) Cibinong Indonesia

**Keywords:** *Adrianichthys*, methods, pelvic brooding, shape analysis, teleost skeleton, trait quantification

## Abstract

Geometric morphometrics (GM) enable the quantification of morphological variation on various scales. Recent technical advances allow analyzing complex three‐dimensional shapes also in cases where landmark‐based approaches are not appropriate. Pelvic girdle bones (basipterygia) of Sulawesi ricefishes are 3D structures that challenge traditional morphometrics. We hypothesize that the pelvic girdle of ricefishes experienced sex‐biased selection pressures in species where females provide brood care by carrying fertilized eggs supported by elongated pelvic fins (“pelvic brooding”). We test this by comparing pelvic bone shapes of both sexes in species exhibiting pelvic brooding and the more common reproductive strategy “transfer brooding,” by using landmark‐free 2D and 3D GM, as well as qualitative shape descriptions. Both landmark‐free approaches revealed significant interspecific pelvic bone variation in the lateral process, medial facing side of the pelvic bone, and overall external and internal wing shape. Within pelvic brooders, the three analyzed species are clearly distinct, while pelvic bones of the genus *Adrianichthys* are more similar to transfer brooding *Oryzias*. Female pelvic brooding *Oryzias* exhibit prominent, medially pointing tips extending from the internal wing and basipterygial plate that are reduced or absent in conspecific males, *Adrianichthys* and transfer brooding *Oryzias*, supporting our hypothesis that selection pressures affecting pelvic girdle shape are sex‐biased in Sulawesi ricefishes. Furthermore, both sexes of pelvic brooding *Oryzias* have overall larger pelvic bones than other investigated ricefishes. Based on these differences, we characterized two reproductive strategy‐ and sex‐dependent pelvic girdle types for Sulawesi ricefishes. Morphological differences between the investigated pelvic brooding genera *Adrianichthys* and *Oryzias* provide additional evidence for two independent origins of pelvic brooding. Overall, our findings add to a better understanding on traits related to pelvic brooding in ricefishes and provide a basis for upcoming studies on pelvic girdle function and morphology.

## Graphical abstract

In our study, we investigate the pelvic girdle shape of eight Sulawesi ricefish species using 2D and 3D landmark‐free geometric morphometrics. Ricefishes evolved two different reproductive strategies, transfer brooding and pelvic brooding, the latter occurring in two distinct ricefish lineages. Our results support the hypothesis of two independent origins of pelvic brooding.

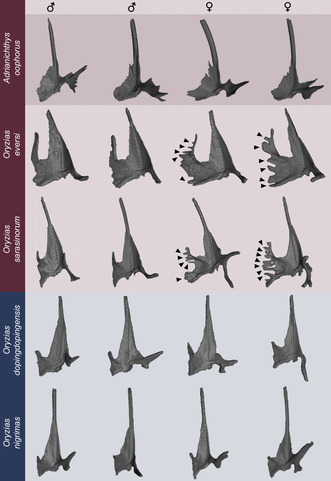

## 1 INTRODUCTION

The vast phenotypic diversity in form and function of organisms reflects adaptation to environmental conditions and the evolution of alternative life‐styles (Losos, 2010; Nosil, 2012; Wainwright, 2007). Variation of morphology, and especially that of structures affecting the interaction of organisms with their environment, is quantified for hypothesis testing in various disciplines such as evolutionary biology (Arnqvist & Danielsson, 1999; Kerschbaumer & Sturmbauer, 2011), developmental biology (Keer et al., 2022; Klingenberg et al., 2001; Sanger et al., 2008), and systematics (Frost et al., 2003; Kergoat & Alvarez, 2008).

Morphometrics provide tools to quantify traits, thus enabling the analysis of form and function (Adams et al., 2004; Cooke & Terhune, 2015; Lauder, 1990). In geometric morphometrics (GM), landmarks are placed on homologous structures to analyze their variation (Bookstein, 1997; Rohlf & Marcus, 1993). GM allow a highly detailed characterization of shape and support various subsequent statistical analyses (Adams et al., 2013).

As an alternative, or addition, to landmark‐based GM, landmark‐free and pseudo‐landmark approaches can describe shapes of morphological or anatomical structures in cases where defining homologous landmarks is challenging or impossible (Boyer et al., 2015; Caple et al., 2017; McCane, 2013; Toussaint et al., 2021), or the landmark distribution is too uneven to capture the shape of interest (Bardua et al., 2019; Goswami et al., 2019; Mitteroecker & Schaefer, 2022; Watanabe, 2018). For example, studies investigating bug wings (Dujardin et al., 2014), bird furcula (Close & Rayfield, 2012), great ape skulls (Rolfe et al., 2021), and marine mammal genitalia (Orbach et al., 2021) show that pseudo‐landmarks provide a valid alternative to landmarks. Landmark‐free and pseudo‐landmark GM approaches have also been used to capture the geometry of complex structures with a focus on their 3D shape (Diamond et al., 2022; Gonzalez et al., 2016; Koehl & Hass, 2015; Pomidor et al., 2016; Porto et al., 2021). A complex morphological structure of acanthomorph fishes is the pelvic girdle (Stiassny & Moore, 1992). Despite the knowledge that pelvic girdles and fins in teleosts can undergo substantial modifications (Don et al., 2013; Yamanoue et al., 2010) or even losses (Bell et al., 1993; Santini & Tyler, 2003), detailed investigations on their form and function are scarce (but see Thieme et al., 2021). Some of the existing evidence suggests that pelvic girdle shapes can be complex and species‐specific (Stiassny & Moore, 1992), making it difficult to determine homologous landmarks.

**FIGURE 1:**
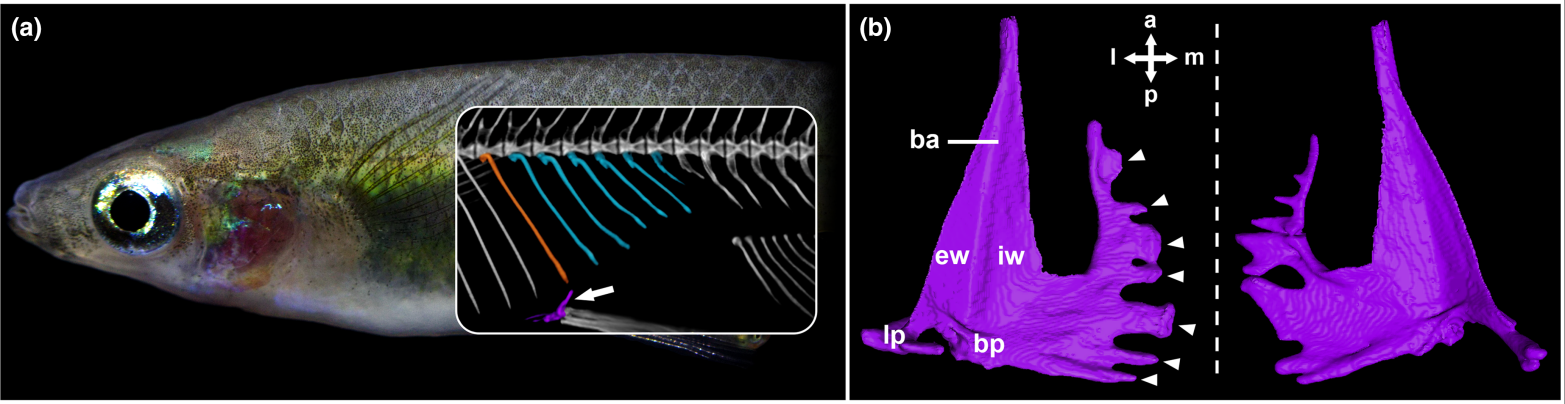
(a) Female *Oryzias eversi* (pelvic brooder) with an X‐ray inlet in the region of the ventral concavity. The rib above the pelvic girdle (purple, arrow) is marked in orange; the following shorter ribs (blue) shape the ventral concavity. (b) Dorsal and close‐up view of the pelvic girdle. Basipterygial arm (ba), basipterygial plate (bp), external (ew) and internal wing (iw) consisting of membranous bone, lateral process (lp) and multiple smaller medial tips (arrow heads) protruding from bp and iw are marked on the left pelvic bone. Dashed line indicates the medial axis. a, anterior; l, lateral; m, medial; p, posterior.

In ricefishes (Beloniformes: Adrianichthyidae), differences of pelvic fin size and thickness were shown to be related to reproductive strategy and sex (Spanke et al., 2021). We thus hypothesize that these differences are also reflected by the shape of the respective pelvic girdle (Figure 1), to which the fins are attached. Ricefishes are a group of small, freshwater fishes distributed over East‐ and South‐East Asia (Hilgers & Schwarzer, 2019; Parenti, 2008; Wittbrodt et al., 2002). The vast majority of ricefishes are “transfer brooders,” as females spawn a small cluster of eggs, which is first attached to their body, but deposited onto submerged substrates shortly after fertilization (Balon, 1975; Iwamatsu et al., 2008; Kottelat, 1990a). A derived reproductive strategy called “pelvic brooding” evolved in species from two distinctly related lineages endemic to the Indonesian island Sulawesi (Gani et al., 2022; Hilgers & Schwarzer, 2019; Kottelat, 1990b; Parenti, 2008). Here, females carry the cluster of fertilized eggs on their ventral side until the embryos hatch. Pelvic brooding comes with a set of adaptations that include a novel egg‐anchoring tissue (Hilgers et al., 2022; Iwamatsu et al., 2008; Schüller et al., 2022), shorter ribs that create a ventral concavity into which the fertilized eggs are placed (Kottelat, 1990b; Popta, 1905; Spanke et al., 2021), and elongated pelvic fins that cover the eggs and ventral concavity (Figure 1). Adaptations to pelvic brooding are thus female‐biased, resulting in a pronounced sexual dimorphism. While a previous study hinted towards conspicuous pelvic girdles in female *Oryzias sarasinorum* (Iwamatsu et al., 2007), it was never investigated in detail. Together with recent findings on ricefish body plan modularity (Flury et al., 2022), these adaptations highlight the special role of the central body region for the rapid and potentially repeated evolution of pelvic brooding.

**FIGURE 2:**
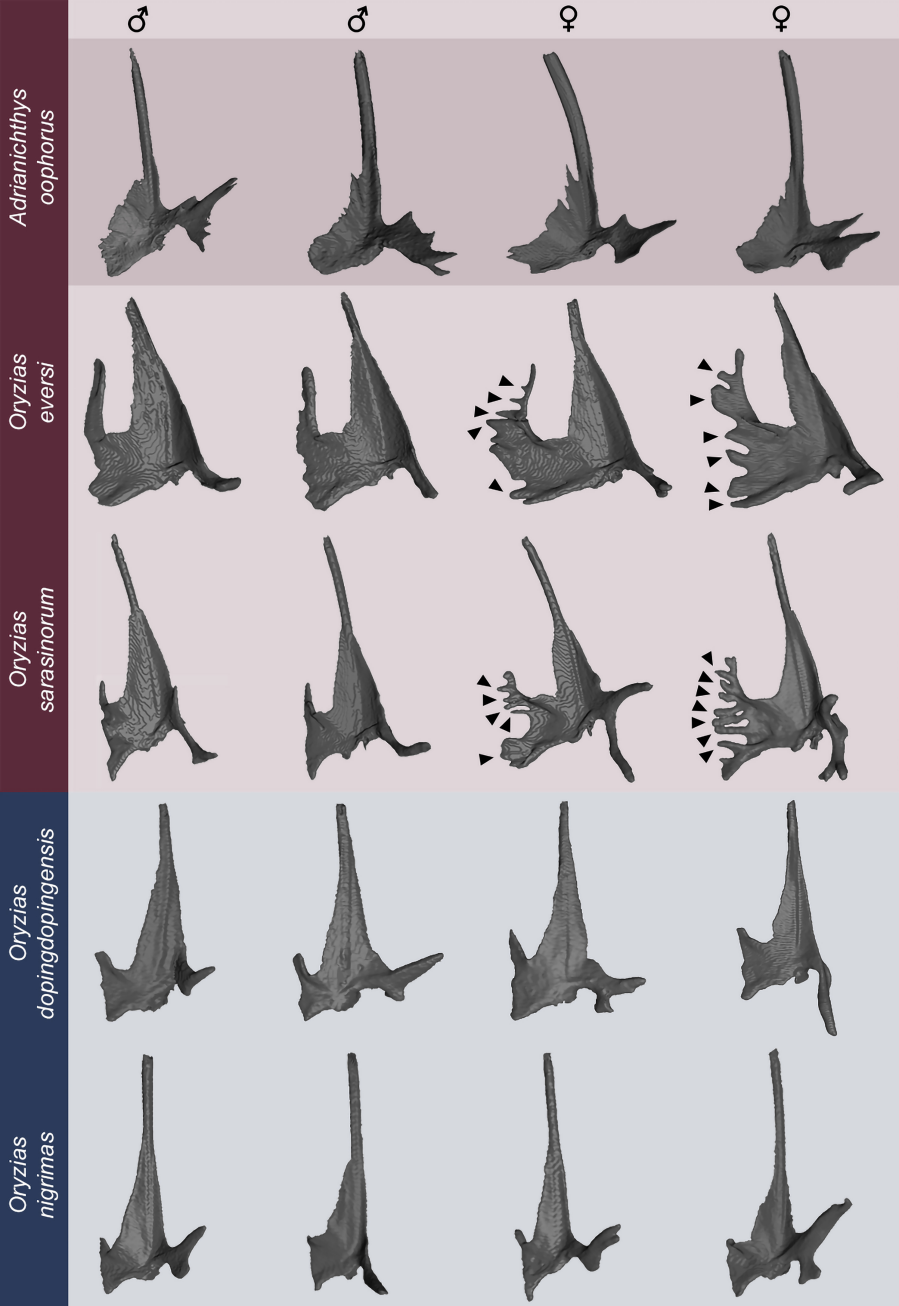
Two‐dimensional representations of the right pelvic bone for male and female ricefishes. Red coloration marks the three pelvic brooding species included in this study, with dark shading representing the *Adrianichthys* lineage. Blue coloration highlights two transfer brooding species. The bones of female *Oryzias* pelvic brooders show medial tips in the form of elongated outgrowths coming from the internal wing, as well as the posterior region of the central part (Figure 1). Male pelvic brooders and transfer brooding specimens show more flattened medial regions of the pelvic bone. Moreover, considerable variation of pelvic bones within same species and sexes can be seen. Top resembles anterior, bottom caudal direction.

Using a comparative framework, we analyzed both sexes of three pelvic brooding (*Adrianichthys oophorus*, *Oryzias eversi*, and *O. sarasinorum*) and five transfer brooding (*O. celebensis*, *O. dopingdopingensis*, *O. matanensis*, *O. nigrimas*, and *O. wolasi*) Sulawesi ricefish species, to investigate whether ricefish pelvic girdles underwent sex‐specific adaptations to pelvic brooding. To describe the pelvic girdle anatomy of ricefishes, we largely followed the nomenclature of Thieme and Moritz (2020). The pelvic girdle of ricefishes consists of paired pelvic bones (basipterygia), composed of a basipterygial arm and a basipterygial plate (together “central part” in Stiassny & Moore, 1992; Figure 1b), respectively. Rays of the pelvic fin attach directly to the basipterygial plate (Figure 1a). An internal and external wing formed from membranous bone are surrounding the central part of the basipterygium (Iwamatsu, 2013; Figure 1b). From the basipterygial plate, a lateral process extends, while medial tips, if present, protrude from the basipterygial plate and the internal wing (Iwamatsu et al., 2007; Parenti, 2008; Figure 1b). Each pelvic bone has a complex, three‐dimensional shape, which can be specimen‐specific (Parenti, 2008; Figures 1b and 2). This variability poses challenges for placing landmarks in 2D and 3D space, as it does not allow for precise and repeatable landmarking of homologous structures (Type‐I landmarking) (Bookstein, 1997; Palci & Lee, 2019; Zelditch et al., 2004). Common shape analysis workflows (e.g., MorphoJ, Checkpoint, or R packages such as geomorph and Morpho), require landmarks at some stage to superimpose specimens in three dimensions (Adams & Otárola‐Castillo, 2013; Bardua et al., 2019; Klingenberg, 2011; Schlager, 2017). We thus utilize three‐dimensional reconstructions of pelvic bones from high‐resolution μ‐CT images, analyzed with landmark‐free outline (2D), as well as surface mesh (3D) GM (GPSA; Pomidor et al., 2016). With this integrated approach, we investigate whether pelvic bones of Sulawesi ricefishes show sex or reproductive strategy‐dependent 3D shapes. We hypothesize that due to the three‐dimensional morphology of the ricefish pelvic bones, the incorporation of 3D geometric morphometrics will provide additional shape information to 2D outline analysis.

## 2 MATERIALS AND METHODS

### 2.1 Study specimens

We strategically chose species in this study so that all six phylogenetic clades of endemic Sulawesi ricefishes are covered (Mokodongan & Yamahira, 2015) and included three pelvic brooding species (Herder et al., 2012; Iwamatsu et al., 2007; Kottelat, 1990b). Per species, five females and five males were studied. The only exception is *O. wolasi*, for which three females and four males were included due to low sample availability. In total, the dataset comprised 77 individuals.

All samples are part of the Ichthyology collection of the Leibniz Institute for the Analysis of Biodiversity Change (LIB), Museum Koenig in Bonn, Germany or the Museum Zoologicum Bogoriense (MZB) in Bogor, Indonesia. *Oryzias dopingdopingensis* were available from a captive population at the LIB in Bonn, Germany. Fish were kept at an 11.5/12.5 light/dark cycle at temperatures between 25°C and 27°C. The fish were euthanized by an overdose of MS‐222 according to the guidelines of the Landesamt für Natur, Umwelt und Verbraucherschutz (§11 Abs. 1Nr 1b, 8a and 8d TierSchG). We provide details of all specimens investigated in Table S1.

### 2.2 μ‐CT imaging

To obtain digital cross‐sections of the pelvic girdle, we created high‐resolution μ‐CT scans of the body region between the pectoral girdle and end of the anal fin for 77 ricefish specimens (Table S1). Prior to scanning, samples were transferred into polypropylene tubes and fully covered with 70% ethanol to prevent shrinkage from dehydration. Samples rested in their tubes for 24 h to settle. To avoid movement during the imaging process, specimens were fixed with small polystyrene blocks. Scans were obtained with a Bruker Skyscan 1272 computer tomographer, operating at 42–52 kV and 140–180 μA. The X‐ray detector was set to an image resolution of 1344 × 2016 pixels (2 × 2 binning), and X‐ray tube values were tuned for each specimen individually in order to adjust for optimal X‐ray transmission intensities. Image pixel size ranged between 7 and 12 μm, depending on the sample's size. We provide details for all scans in Table S2. The computation of cross sections from the μ‐CT images and subsequent cross section processing was carried out using Bruker's NRecon and DataViewer software. All μ‐CT scans are part of the collection of the LIB.

### 2.3 Data collection

For the two‐ and three‐dimensional shape analyses, we used surface renders of the pelvic bones from 77 ricefish specimens generated in Amira 6.5.0 (Thermo Fisher Scientific). Surface renders were created by choosing a gray‐value range that selected the pixels resembling the left and right pelvic bone and their fin rays in the previously obtained μ‐CT cross sections (see above). To mark these pixels across all slices, the “same material” propagating algorithm of the quick selection tool was used. Pixels with similar gray‐values connected to each other in adjacent slices were selected, thus masking the entire left and right pelvic bone in the image stack. In a control step after this semi‐automated segmentation, imperfect selections were adjusted manually to ensure the final volume render precisely matched the pelvic bones. Finally, the selected pixels were transferred into three‐dimensional renderings using the “surface render” module of Amira and exported as ascii PLY files. Since four specimens had fractures in the left pelvic bone, but only one specimen had a fractured right pelvic bone (*O. dopingdopingensis*; ICH‐126857), we excluded that specimen and conducted the morphometric analyses with the right pelvic bone of the 76 remaining specimens. Pelvic bone contour normalization and PC scores for 2D morphometrics were estimated in SHAPE (Iwata & Ukai, 2002; v. 1.3), while ordination coordinates for 3D morphometrics were calculated in GPSA (Pomidor et al., 2016; v. 20200722).

### 2.4 2D outline shape analyses

To investigate whether pelvic bone morphology correlates with reproductive strategies and sex in Sulawesi ricefishes, we conducted a two‐dimensional Fourier outline shape analysis (Iwata & Ukai, 2002). This method uses sine and cosine terms (harmonics) to describe the closed outline of a shape (Kuhl & Giardina, 1982). From one harmonic representing a simple ellipse, adding harmonics allows the outline to become more complex, representing the desired shape (Crampton, 1995). To acquire 2D representations of the pelvic bones, we transferred 3D pelvic bone surface renders into 2D images in the following manner: Each 3D surface was manually rotated in Amira so that the tip of the basipterygial arm was aligned vertically in the YZ (lateral) viewing plane (Figure S1). Simultaneously, the medial part of the pelvic bone was horizontally oriented in the XY (anterior–posterior) viewing plane, to obtain a positioning to which all pelvic bones could be aligned (Figure S1). After orientating the pelvic bones along these two axes, two‐dimensional snapshots in the XZ (dorso‐ventral) viewing plane were taken to obtain images with the dorsal side of the pelvic bone facing upwards. Since fin rays are no rigid structures suited for homologous shape comparison (e.g., Krieger, 2010; Palci & Lee, 2019), these were manually removed from the 2D images in Affinity Photo (v. 11.0). Outline shapes were generated from the final images using the “SHAPE” (v. 1.3) software package and ChainCoder module (Iwata & Ukai, 2002). In ChainCoder, pelvic bone snapshots were binarized into black (pelvic bone) and white (background) color images using a histogram function. Then, a chain code (Freeman, 1974) from the contrast of the binary image resembling the outline of the pelvic bone was generated and lastly stored in a text file for subsequent statistical analyses. For 2D outline analyses, elliptic Fourier descriptors (EFDs) (Kuhl & Giardina, 1982) were calculated based on 26 harmonics using the “Chc2Nef” module of SHAPE and the chain code text file. The EFDs were normalized in “Chc2Nef” by using the longest radius method. PC scores were then estimated based on the normalized EFDs in the “PrinComp” module of SHAPE.

### 2.5 3D pelvic bone shape analyses

Since pelvic girdles of ricefishes might exhibit 3D shape differences between sexes and reproductive strategies that are not captured by 2D outline analysis, we additionally employed landmark‐free surface geometric morphometrics. To compare the three‐dimensional pelvic bone shape of ricefishes, we used 76 surface renders obtained from semi‐automated segmentation in Amira. Shape analyses were carried out using the JAVA software package “Generalized Procrustes Surface Analysis” (GPSA, v. 20200722 provided by B. j. Pomidor upon personal request) (Pomidor et al., 2016).

To exclude non‐rigid structures during shape comparison, we manually removed fin rays from the initial Amira meshes in Blender (Blender Development Team, v. 3.0). The surface renders were then remeshed in Blender using the “sharp remesh modifier” with an octree depth of 10 and scale factor of 0.6. This resulted in meshes with approximately 2 million faces and ensured an even distribution of vertices and triangles over the mesh. Since GPSA uses an iterative closest point (ICP) algorithm for the surface analysis, a uniform distribution of vertices is desired for 3D landmark‐free shape analysis (Pomidor et al., 2016). Moreover, the remeshing process closed holes in the pelvic bone meshes that originated from imperfections in the segmentation procedure and removal of fin rays. Holes that still remained after remeshing were closed using the “close holes” filter of Meshlab (Cignoni et al., 2008).

To investigate whether a higher number of faces provides better alignment for complex morphological shapes when using GPSA, the face number of the remeshed pelvic bones was reduced and equalized in Blender using the “decimate” modifier. We created three datasets, containing the right pelvic bone of 76 specimens with either (1) 200,000, (2) 500,000 or (3) 800,000 faces.

### 2.6 Landmark‐free surface analysis

Analyzing 3D shapes in GPSA requires surfaces to be superimposed to a prototype using a modified ICP algorithm (Chen & Medioni, 1991; Pomidor et al., 2016). As prototype selection could impact the analysis, it is crucial to carefully select the specimen that serves as prototype. It is recommended to select a specimen of intermediate shape, ideally covering the morphological variation present in the dataset (Pomidor et al., 2016). After superimposition, a Procrustes surface metric (PSM) is calculated based on the distance between points of the mean shape (prototype) and each specimen in the dataset, likewise to generalized Procrustes analysis and the Procrustes distance (Gower, 1975; Pomidor et al., 2016).

We preselected two female individuals (MZB25203, MZB25205; both *Adrianichthys oophorus*) based on the abovementioned characteristics. To determine the number of faces and the more suitable prototype for our dataset, we applied two criterions: (1) The total number of specimens aligned to the two selected prototypes during the Procrustes analysis and (2) a disparity measure describing how close specimens of the same species cluster in the morphospace. For the disparity measure, we selected “ranges” that capture the absolute ranges of standardized PC scores across PC dimensions and the “convex hull surface” spanning the morphospace (Guillerme, 2018). We screened for prototype and dataset combinations that maximized ranges at minimal convex hull surfaces. This approach is based on the premise that specimens of the same species should align more closely in the morphospace. The convex hull disparity measure provides information about the overall size of the morphospace. We propose that larger morphospaces with less interspecimen variation are indicative of noise captured in the PC scores, prone to outliers. On the contrary, overall smaller morphospaces with higher interspecimen variation point toward more distinctively separated specimens.

### 2.7 Statistical analyses

All statistical analyses were carried out in R version 4.1.1. We used the R package “PCDimensions” to estimate significant PCs for the shape analyses (2D and 3D) based on a broken stick model (Jackson, 1993; Wang et al., 2018). To test for significant differences in pelvic bone shape between species and sexes, the R package “vegan” (Oksanen et al., 2022) was used to run PERMANOVA models on the reduced sets of 2D and 3D geometric morphometric PCs with 9999 permutations. Tests were carried out including “reproductive strategy,” “sex,” and “species” as predictor variables and standard length as covariate. Since we expect effects to be specifically related to the brooding sex, we also allowed an interaction between “sex” and “reproductive strategy” (transfer brooding or pelvic brooding) in the model.

To identify the best‐fitting prototype in 3D shape analysis for the different datasets (200,000, 500,000, 800,000 faces), both prototypes (see section above) were tested by Procrustes aligning specimens to them (Pomidor et al., 2016). Since our pelvic bone samples are complex in shape and show considerable morphological variations, we checked alignment consistency in GPSA by repeating the process for each prototype and dataset combination five times. Dataset‐prototype combinations that showed the smallest number of misaligned specimens were then subjected to the disparity analysis. As disparity measure, we selected the indices “range” and “convex hull” of the R package “dispRity” (Guillerme, 2018). We addressed dataset comparability by normalizing PC scores obtained from GPSA into z‐scores using the mean and standard deviation of each individual PC axis, followed by the abovementioned dimensionality reduction (Fruciano et al., 2017; Pomidor et al., 2016). For the disparity index (“range,” “convex hull surface”) calculation, we prepared a single matrix containing z‐transformed PC scores of dataset‐prototype combinations that all aligned the highest number of pelvic bones. This matrix was subset into groups corresponding to the dataset‐prototype combinations. A bootstrap factor of 5000 was chosen and t‐statistics comparing the indices between groups were carried out in “dispRity” with Bonferroni correction for multiple testing.

### 2.8 Effect of phylogenetic signal on pelvic bone shape

To estimate the impact of relatedness on differences in pelvic bone shape between reproductive strategies, we conducted a phylogenetic least square (PGLS) analysis based on a published ricefish phylogeny (Mokodongan & Yamahira, 2015). Recent molecular studies investigating ricefish evolution revealed highly congruent phylogenies, but differed in sister group relationships of Sulawesi *Oryzias* species (Flury et al., 2023; Gani et al., 2022; Mokodongan & Yamahira, 2015). We pruned the phylogeny using the R package “caper” (Orme et al., 2012) to only contain the eight species included in this study and took a conservative approach for the PGLS analysis by defining a polytomy for the ambiguous node between the clade including the pelvic brooding *Oryzias*, Lake Malili and Lake Poso *Oryzias* species (Flury et al., 2023). Each sex was tested separately after calculating species‐specific means of the PC scores. We also included a male‐to‐female ratio to test for differences in the degree of sexual dimorphism between reproductive strategies. Six individual models were calculated using the pgls function of “caper” (Orme et al., 2012) with PC1 to PC6 as dependent variable, while predictor variables included “reproductive strategy” and “standard length.” The PGLS analysis calculates the scaling parameter λ to assess the degree of phylogenetic independence of the data (Pagel, 1997). This value has a range of 0 (low signal) to 1 (strong signal) and is calculated based on likelihood ratio tests (Orme et al., 2012). We chose a conservative approach and set λ to 1 if it did not significantly differ from it. Like this, we can get an estimate accounting for strong phylogenetic signal on our predictor variables.

### 2.9 Correlation of 2D and 3D dataset

By cross‐referencing the contour shape deformations obtained from SHAPE and surface difference information gathered in GPSA, a more complete view of the pelvic bone morphology can be inferred. Therefore, we compare the shape changes visualized over principal components in wireframe graphs (2D outline) with heat maps showing areas of morphological differences in pelvic bone structure (3D surfaces).

In the landmark‐free 2D shape analyses of the pelvic bone, changes in structural information are not visible, whereas in the 3D landmark‐free geometric morphometrics approach, fine‐structured shape variations that are not present in the prototype get lost and thus are not represented in the results. By combining both approaches, method‐based limitations in visualization of shape changes can be compensated for, which provides a more complete understanding of complex morphological structures such as pelvic girdles.

## 3 RESULTS

### 3.1 2D shape variances of transfer and pelvic brooding pelvic bones

Qualitative two‐dimensional shape comparisons of pelvic bones from transfer (*O. celebensis*, *O. dopingdopingensis*, *O. matanensis*, *O. nigrimas*, *O. wolasi*) and pelvic brooding (*A. oophorus*, *O. eversi*, *O. sarasinorum*) ricefishes revealed species‐ and sex‐specific morphologies with intraspecific variation (Figure 2). Female individuals of pelvic brooding *Oryzias* species showed medial tips, which emerged from the internal wing and basipterygial plate. These tips exhibited considerable variation between individuals (Figure 2). Pelvic bones of conspecific males displayed strongly reduced or no medial tips, and medial tips were also absent in both sexes of transfer brooding species, as well as the pelvic brooding *Adrianichthys* lineage (Figure 2). Shape variations between individuals of the same species and sex were present in the external and internal wing, as well as the lateral process, but individual variations were more pronounced in the lateral process. Lateral processes of the same species and sex could for example point in different directions (more caudal or anterior; Figure 2, e.g., *O. nigrimas* males), or show varying bifurcations (Figure 2, e.g., *O. sarasinorum* females).

### 3.2 2D geometric morphometrics of pelvic bone shape

**FIGURE 3:**
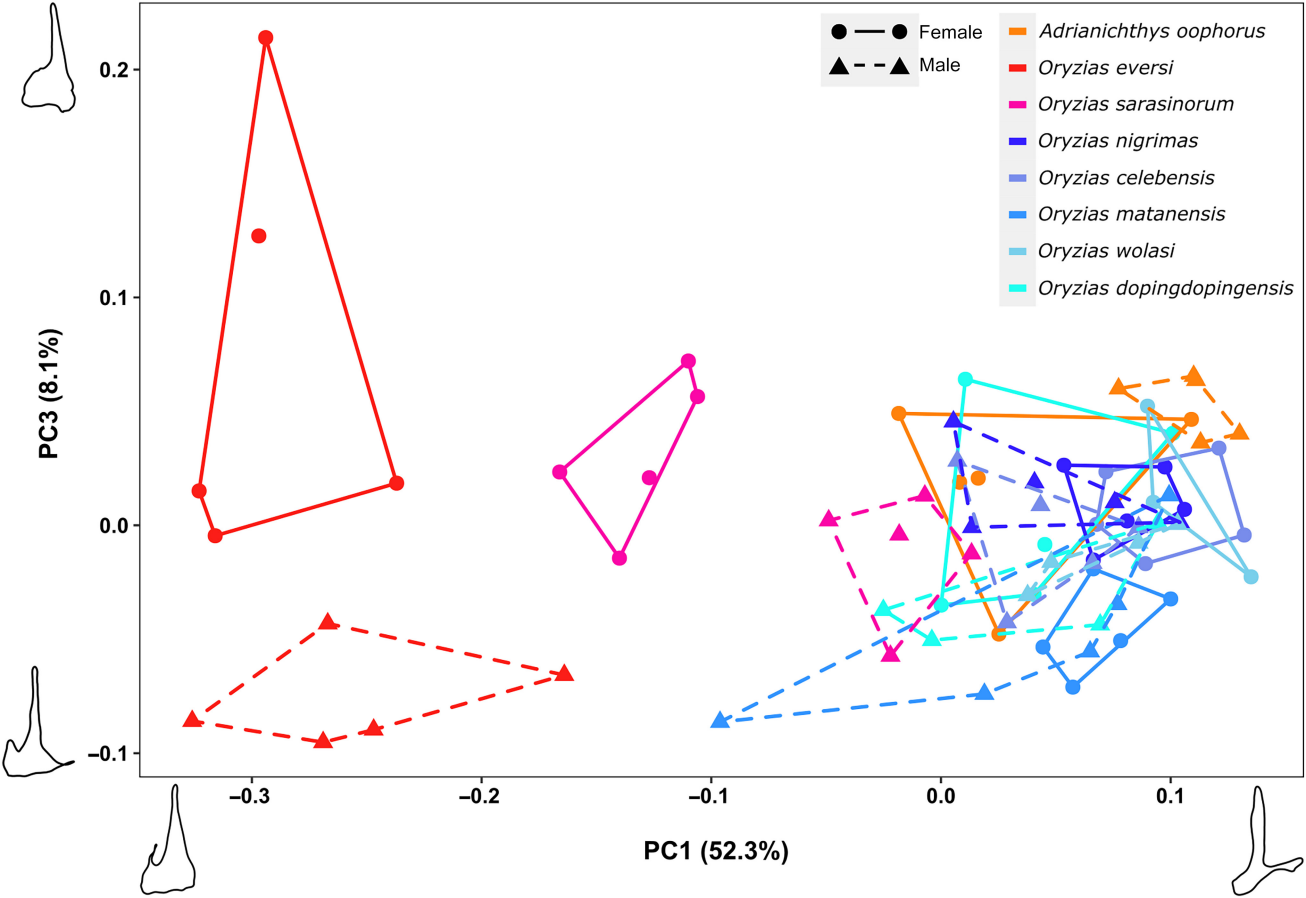
Principal component plot visualizing shape differences in the pelvic bones of Sulawesi ricefishes. Data is based on 2D elliptic Fourier outline analysis of pelvic bones. Convex hull border and point shape indicate sex (dot, full line: females; triangle, dashed: males). The plot shows that females of pelvic brooding ricefishes cluster individually. Figure S2A shows PC score boxplots per sex and species. Differences in their shape attribute to a larger internal wing and the presence of medial tips (PC1) in *Oryzias* pelvic brooders compared to *Adrianichthys*. Moreover, a sexual dimorphism in pelvic brooding species is visualized over PC1 (*A. oophorus*, *O. sarasinorum*) and PC3 (*O. eversi*) (orange, pink and red colored symbols). PERMANOVA analyses on PC axes 1–6 confirmed that reproductive strategy and sex, as well as their interaction, are significant predictors for pelvic girdle shape (*p* < .001).

After dimensionality reduction, meaningful principal components 1–6 accounted for 84.4% of the shape variation in the 2D dataset; all following individual axes contained <4% of the variation (Table S3). Shape differences between ricefish species and sexes were primarily captured by PC1 and PC3 (Figure 3). PC1 depicts differences of the medial tips and internal wing, while PC3 shows variation of the lateral process and the transition zone of the basipterygial arm to the surrounding wings. We excluded PC2, since it shows variation in the caudal location of the pelvic bone where the fin rays attach, potentially impacted by the manual fin‐ray removal.

We observed “species” to be a significant predictor (PERMANOVA: *p* = .0001, df = 6; *R* ^2^ = .4, *F* = 13.78) for the variance captured in the 2D PC scores, generally pointing toward shape differences among the investigated ricefishes. The explanatory variable “reproductive strategy” was also highly significant (PERMANOVA: *p* = .0001, df = 1, *R* ^2^ = .23, *F* = 48.37), revealing differences between pelvic and transfer brooding individuals (Figure 2 and Table S4). The predictor “sex” was not significant (PERMANOVA: *p* = .07, df = 1; *R* ^2^ = .01, *F* = 2.38); however, the interaction between “reproductive strategy” with “sex” was highly significant (PERMANOVA: *p* = .0003, df = 1, *R* ^2^ = .04, *F* = 8.42), pointing toward sex‐specific differences among pelvic and transfer brooding species (Figure 2 and Table S4). Pelvic brooding species *A. oophorus*, *O. eversi*, and *O. sarasinorum* were largely distinguished from each other by their medial region of the pelvic bone (PC1), while the sexes of *O. eversi* and *O. sarasinorum* are further separated on PC1 and PC3 by lateral process shape (Figure 2). Differences between females of both reproductive strategies persisted, even when setting λ to 1 (PGLS_PC1_: *p* = .0005, df = 5, *R* ^2^ = .0.95, *F* = 50.42; Table S8). For male specimens, PGLS models for PC1 estimated low phylogenetic signal significantly different from 1 (Table S8) and a significant effect of the “reproductive strategy” predictor (PGLS_PC1_: *p* = .022, df = 5, *R* ^2^ = .71, *F* = 6.23; Table S8). Furthermore, the PCA comparing outline shapes showed that *A. oophorus*, contrary to the *Oryzias* pelvic brooders, form a cluster within the morphospace of transfer brooding species (Figure 3, orange symbols).

### 3.3 3D mesh size and prototype can impact alignment

In order to ensure an optimal data basis for the 3D surface analysis in GPSA, we prepared six datasets, based on the combinations of two GPSA prototypes and three surface model mesh sizes. After selecting two *A. oophorus* specimens from our dataset (MZB25203 and MZB25205) for the alignment of all specimens using a Procrustes surface metric (Pomidor et al., 2016) and combining them with meshes having 200,000, 500,000, and 800,000 faces, we observed that face number and prototype combinations impacted the alignment process (Table S5). Differences included total number of aligned specimens, as well as mesh orientation after the alignment process. The maximum number of well aligned specimens was 74 out of 76, misaligning one *O. dopingdopingensis* (ICH‐126862) and one *O. eversi* (ZFMKICH121935). Mesh size and prototype combinations 200,000:MZB25203, 500,000:MZB25203, 800,000:MZB25203 and 500,000:MZB25205 aligned these 74 specimens equally well. Combinations of 200,000 and 800,000 face meshes with the MZB25205 prototype performed worse, misaligning one (*O. eversi*, ZFMKICH121936) and two (*O. eversi*, ZFMKICH121936; *O. wolasi*, ICH‐126863) additional specimens, respectively (Table S5).

### 3.4 Disparity metrics differ depending on mesh‐size and prototype

The disparity metrics (DM) on z‐transformed PC scores (axes 1–6) show that prototype selection and number of faces in 3D meshes impact the morphospace structure with respect to convex hull surface area and PC score range. Lower face numbers in case of the 200,000 face meshes paired with prototype MZB25203 lead to a significantly smaller convex hull surface metric (DM: 877.58), compared to the 800,000 (DM: 882.6) and 500,000 (DM: 937.52) face meshes aligned with prototype MZB25203 (all comparisons *p* < .0001; Figure S3A and Tables S6 and S7). The largest convex hull surface volume was observed using the MZB25205 prototype and meshes with 500,000 faces (DM: 1126.48; all comparisons *p* < .0001; Figure S3A and Tables S6 and S7).

Additionally, significant differences between datasets in the sum of ranges metric were observed. The 200,000 faces dataset paired with the MZB25203 prototype showed significantly higher median and quartile scores (Median: 34.9757) compared to the 500,000 (Median: 33.4945) and 800,000 (Median: 31.0966) face datasets aligned with MZB25203 (all comparisons *p* < .0001; Figure S3B and Tables S4 and S5). The highest median value was observed in the 500,000 face dataset aligned with prototype MZB25205 (Median: 35.4145, all comparisons *p* < .0001; Table S7); however, its 0.25–0.75 quartile showed a lower range (quartile range: 3.64) compared to the 200,000:MZB25203 dataset (quartile range: 4.39). Based on these results, we selected the dataset with 200,000 faces aligned with prototype MZB25203 (smallest convex hull, largest ranges; Table S6) for the final shape analysis in GPSA.

### 3.5 3D geometric morphometrics of pelvic bone shape

**FIGURE 4:**
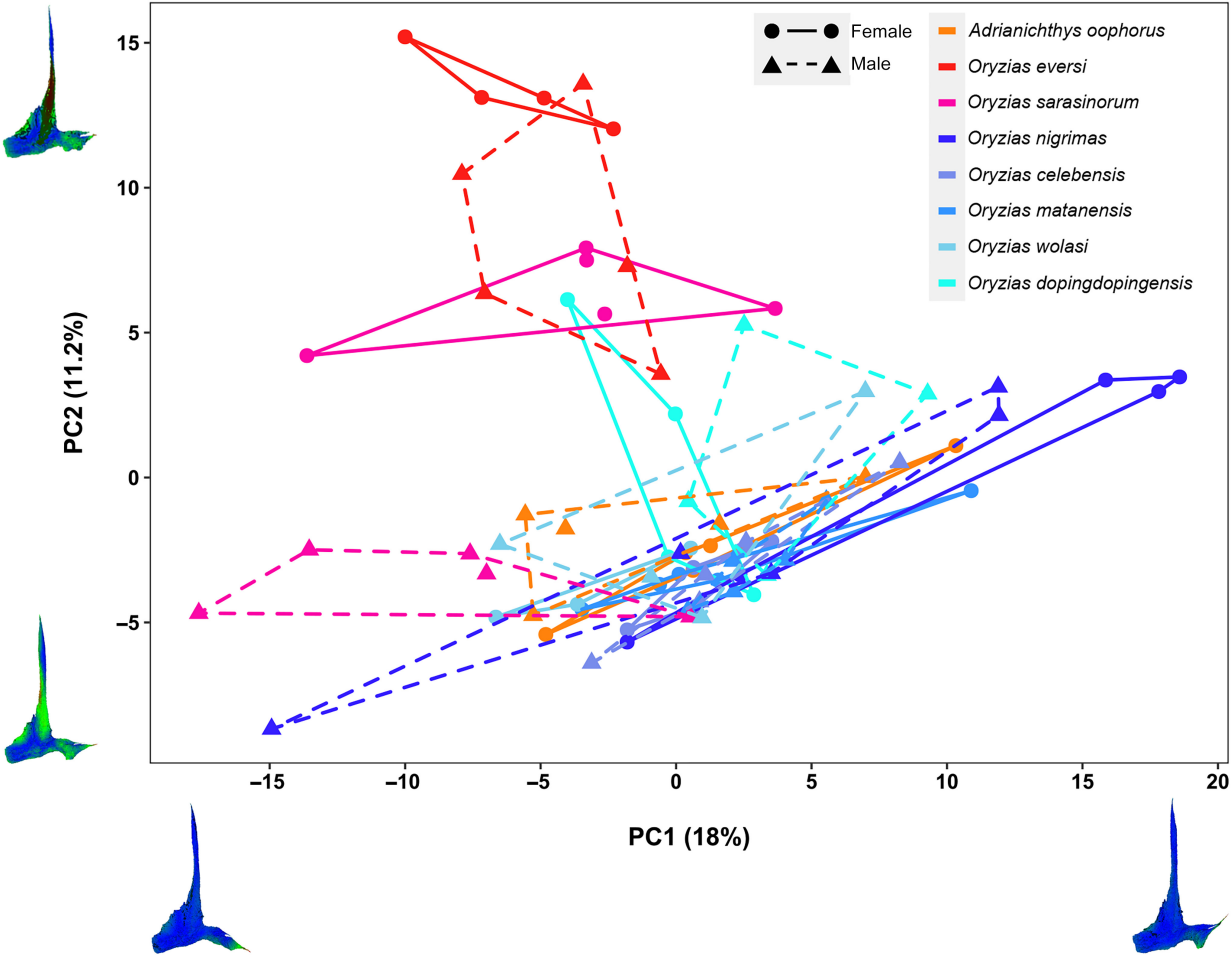
Principal component plot visualizing shape differences in the pelvic bones of Sulawesi ricefishes. Data is based on 3D generalized Procrustes surface analysis of pelvic bones. Convex hull border and point shape indicate sex (dot, full line: females; triangle, dashed line: males). The ordination separates females of the three pelvic brooding lineages (red colored symbols) and reveals that pelvic bones of *Oryzias* pelvic brooders are more similar to each other than *Adrianichthys* in the medial part of the pelvic bone (PC2, medial tips, larger internal wing). Moreover, the pelvic bone shape of the pelvic brooding *Adrianichthys* lineage is more similar to transfer brooding species (blue colored symbols), showing a smaller internal wing and no medial tips. PERMANOVA analyses (axes 1–6) reveal that ‘reproductive strategy’ and the interaction of ‘reproductive strategy’ with ‘sex’ are significant predictors for pelvic bone shape (*p* < .001). Figure S2B shows ordination score boxplots per sex and species. Shape changes are depicted using heat maps on mean shapes of the pelvic bone. Color on pelvic bone shapes resembles low (blue) to stronger (green, red) variation in the respective regions of the pelvic bone (see Figures S4 and S5).

Comparing three‐dimensional pelvic bone shapes of Sulawesi ricefishes using generalized Procrustes surface analysis (GPSA) revealed sex‐specific differences and variation based on reproductive strategy (pelvic or transfer brooding). After dimensionality reduction, PC axes 1–6 accounted for 51% of shape variation captured in the 3D dataset, while remaining individual axes contained <4% variation (Table S3). The GPSA ordination captured three major shape variances in ricefish pelvic bones on PCs 1–3. They depict lateral process orientation, the shape of the internal wing and basipterygial plate with protruding medial tips, as well as the basipterygial arm (Figure 4 and Figures S4 and S5). Similar to 2D shape analysis, the predictor “species” was highly significant (PERMANOVA: *p* = .0001, df = 6; *R* ^2^ = .269, *F* = 4.99), which points toward general pelvic bone shape differences among ricefish species. PERMANOVA also revealed that “reproductive strategy” (*p* = .0001, df = 1, *R* ^2^ = .116, *F* = 12.91), as well as the interaction of “reproductive strategy” and “sex” (*p* = .01, df = 1, *R* ^2^ = .03, *F* = 3.10), explain significant differences in 3D pelvic bone shape. The predictor “sex” was non‐significant (PERMANOVA: *p* = .24, df = 1, *R* ^2^ = .012, *F* = 133). The PGLS analysis for females estimated low phylogenetic signals, except for PC4. However, even under the assumption of high phylogenetic signal, significant differences between female pelvic and transfer brooding individuals persisted (Table S8).

For male specimens, the phylogenetic models estimated low phylogenetic signal in PCs 1, 2 and 6 with a significant effect of “reproductive strategy” in PC1 (Table S8).

Pelvic brooding females of *O. eversi* and *O. sarasinorum* separated from other females on PC2, pointing toward a thicker basipterygial arm and wider internal and external wings in the central region of the pelvic bone in these specimens (Figures 3 and 4, red and pink data points). Additionally, PC2 depicts variation on the medial and anterior ranging parts of the pelvic bone. Deformation plots of the GPSA analysis showed that *O. eversi* and *O. sarasinorum* females have overall larger internal and external wings and differ from other specimens in having larger girdles in the medial facing part of the pelvic bone (Figure 1, medial tips; Figure 4).

Pelvic bones of male *O. eversi* specimens were intermediate between females of *O. eversi* and *O. sarasinorum*, thereby separating from other male ricefishes (Figure 4, red triangles). Pelvic bone shape of male *O. sarasinorum* was more similar to male and female transfer brooding individuals regarding PC2 (internal wing and basipterygial plate shape), separating toward the lower left of the PC plot (Figure 4, pink triangles). Individuals of *A. oophorus* showed less extreme sex‐specific differences and grouped within the large cluster of transfer brooding ricefish species (Figure 4, orange symbols).

## 4 DISCUSSION

We apply two landmark‐free approaches, Fourier outline (2D) and generalized Procrustes surface (3D) GM, to describe the complex shape of Sulawesi ricefish pelvic bones challenging the placement of traditional landmarks. Particularly in the 3D shape analysis, we found that 3D mesh size and prototype selection (Pomidor et al., 2016) can impact the alignment of the specimens (Table S5), which adds to other studies emphasizing a well‐thought‐out preparation of 3D landmark‐free or point‐cloud‐based GM datasets (Gonzalez et al., 2016; Mitteroecker & Schaefer, 2022; Pomidor et al., 2016; Porto et al., 2021). Nevertheless, by accounting for 3D mesh size and prototype selection in our 3D dataset, both landmark‐free GM approaches similarly differentiate ricefish pelvic bones according to reproductive strategy and generate analogous morphospaces (Figures 3 and 4). The most pronounced differences in pelvic bone shape can be found in internal and external wings along the basipterygial arm and in the medial part of the pelvic bone (Figures 1 and 2). In female pelvic brooding *Oryzias*, the shape of the internal and external wings around the basipterygial arm grows wider from anterior to posterior and is therefore larger compared to other ricefishes (Figure 3, PC1; Figure 4, PC2; Figure S4). Most conspicuous is the medial region of the pelvic bone, as it separates *Adrianichthys* from pelvic brooding *Oryzias* due to the medially ranging tips present in female pelvic brooding *Oryzias* (Figures 2 and 3, PC1; Figure 4, PC1, PC2; Figure S4). *Adrianichthys* pelvic bone shapes are more similar to those of transfer brooding ricefishes (Figures 3 and 4), despite the fact that other traits related to pelvic brooding morphologically differentiate females of all to date investigated pelvic brooding species from transfer brooding ricefish species (see following section).

### 4.1 2D and 3D pelvic bone morphometrics separate pelvic brooding ricefish lineages

Traits such as brooding duration, length of ribs dorsal of the pelvic fins (Figure 1), volume of the caudal body region, as well as pelvic fin‐ray length and thickness are different in females of pelvic brooding compared to females of transfer brooding ricefish species (Gundo et al., 2016; Herder et al., 2012; Iwamatsu et al., 2007; Kottelat, 1990b; Parenti, 2008; Spanke et al., 2021). Due to the structural integration of the pelvic girdle with the pelvic fins, we hypothesized that the observed differences in pelvic‐fin length and fin‐ray thickness would be reflected in the shape of the pelvic bones. This was, however, only partly confirmed. Generally, pelvic brooding species span a wider morphospace in 2D and 3D pelvic bone shape comparisons than transfer brooding ricefishes due to prominent variation between *O. eversi* and *A. oophorus* (Figure 3, red, orange, pink symbols; Figure 4, red, orange, pink symbols). Females of *O. sarasinorum* grouped in between these two extremes, while *O. sarasinorum* males clustered more closely with both sexes of *Adrianichthys* than their conspecific females (Figures 3 and 4). Both approaches revealed that females of all pelvic brooding ricefishes form distinct clusters and separate from their corresponding males (Figures 3 and 4 and Table S4). This sexual dimorphism was more pronounced in *Oryzias* species than in *Adrianichthys oophorus* (Figures 3 and 4; red, orange, pink symbols). As both sexes of *Adrianichthys* grouped within transfer brooding species, there is no common morphological pattern separating pelvic bone shapes of the two reproductive strategies. Although the shape of the pelvic bone is sexually dimorphic in pelvic brooding species (Figure 3, PC1), these differences are overridden by even greater interspecific differences (Figure 4, PC2).

There is growing evidence that pelvic brooding evolved in parallel within Sulawesi ricefishes. The two lineages in which pelvic brooding species occur (*Adrianichthys* and one monophyletic group of Central Sulawesi *Oryzias* species) split ~15 mya (Mokodongan & Yamahira, 2015) with no signs of introgression evident between them (Flury et al., 2023; Montenegro et al., 2022). Furthermore, specific differences in pelvic‐fin morphology and differences in the allocation of body cavity volume anterior of the pelvic girdle between *Adrianichthys* and *Oryzias* emphasizing lineage‐specific trait variations (Montenegro et al., 2022; Spanke et al., 2021). The distinct morphological pelvic bone shape of *A. oophorus* adds to these lineage‐specific traits within pelvic brooding ricefishes and is in line with a parallel evolution of pelvic brooding in *Adrianichthys* and *Oryzias*.

### 4.2 Potential pitfalls of landmark‐free 3D GM

In ricefishes and other Beloniformes, lateral processes growing from the pelvic girdle along the body wall (Parenti, 2008), as well as asymmetric pelvic bones (Iwamatsu, 2013; Figure 1) were described. These create complex, three‐dimensional structures. In order to relate specific pelvic bone morphologies with ricefish reproductive strategy or sex it was thus crucial to account for this three‐dimensionality. However, landmark‐free (like GPSA; Pomidor et al., 2016) or point‐cloud based 3D approaches (like ALPACA; Porto et al., 2021) require the selection of a prototype or template to align shapes to each other. This is especially challenging when—like in our case—the structure is highly complex (e.g., medial tips and bifurcated lateral processes) and shows strong intra‐ and interspecific shape variations (Pomidor et al., 2016; Porto et al., 2021). Here, it is unavoidable that the prototype misses certain shape information. Our prototype for 3D analyses was the pelvic bone of MZB25203 (*Adrianichthys*) that lacks some of the variation present in the medial region of the pelvic bone (Figure S2, red areas; Figure S4, PC2, green areas), which makes an interpretation of the results in this specific area challenging. By employing GPSA, we could capture the 3D orientation of the lateral process and curvature of both (external and internal) wings and the basipterygial plate in the complex pelvic girdle structure. The integration of both (2D and 3D) GM techniques along with qualitative descriptions then allowed us to thoroughly investigate the shape differences in pelvic girdles of Sulawesi ricefishes.

### 4.3 Pelvic bone shape variation in Sulawesi ricefishes

Based on qualitative (Figure 2), as well as 2D and 3D GM analyses (Figures 3 and 4), the lateral pelvic girdle processes (Figure 1) of ricefishes analyzed in this study showed strong intra‐ and interspecific variation (Figures 1 and 2 and Figure S2). Individuals of similar species and sexes differed in lateral process shape in both GM analyses, though this was especially prominent in the 3D approach (Figure 4, PC1). This variability, that we also see in our descriptive data (Figure 2), likely results from bifurcations of the lateral process, which was also described earlier for ricefishes and other Beloniformes (Parenti, 2008). The lateral process of Atherinomorphs is further aligned with a dorsally adjacent rib and in some species even connected to it by a ligament (Parenti, 1993), which might lead to additional variation in the shape of this process. Due to this high individual variability, we exclude the lateral process as a classification criterion to group the herein analyzed pelvic bones of Sulawesi ricefishes.

Pelvic bone shapes of Sulawesi ricefishes can, based on the eight species investigated here and applying a purely descriptive approach, be grouped into two main categories (based on the internal wing and medial tips):

(1) Pelvic bone medially flattened with a smaller internal wing, lacking medial tips. *Adrianichthys* specimens, transfer brooding females and all investigated males belong to this category. (2) Pelvic bone with a larger internal wing and female‐specific medial tips. The second group includes only pelvic brooding females of *O. eversi* and *O. sarasinorum* (Figures 2, 3, 4). This basic categorization of pelvic bone morphologies does not show a general separation by reproductive strategy and sex, opposing the pattern found in the ribs forming the ventral concavity (Spanke et al., 2021) or pelvic fin length (Gundo et al., 2016; Herder et al., 2012; Kottelat, 1990b).

### 4.4 Pelvic bone function and outlook

In most teleosts, the pelvic girdle serves as an attachment point for the fin rays and the corresponding musculature (Yamanoue et al., 2010). Larger pelvic bones have been shown to develop in response to increased stresses, like the perching behaviors of cyprinids and sisorids (Saxena & Chandy, 1966), during walking‐like movement of cavefishes (Flammang et al., 2016) or hillstream loaches (Crawford et al., 2020) and the sucking mechanism in climbing gobies (Budney & Hall, 2010). The larger internal wing of *O. eversi* and *O. sarasinorum* could act in a similar manner, providing more support for their elongated and thickened pelvic fin‐rays (Spanke et al., 2021). Additionally, an overall larger pelvic bone would provide more area for the muscles that attach to the fin rays. Since the eggs in pelvic brooding ricefishes are covered by the females' pelvic fins, larger muscles could provide additional force to tug the eggs into the ventral concavity (Figure 1). Especially, interesting in this regard are the conspicuous medial tips of *O. eversi* and *O. sarasinorum*. Muscles attaching over the medial side of the pelvic bone show their largest extend in the posterior region (Yamanoue et al., 2010). This coincides with the position of the medial tips on pelvic girdles in females of *O. eversi* and *O. sarasinorum*, potentially providing more surface area for attaching musculature (Figure 2).

Pelvic brooding ricefishes carrying their eggs while covering them with elongated pelvic fins likely require adaptations of the pelvic girdle, fin rays and muscles to compensate for added weight and an altered body shape (egg‐cluster) when swimming. Since pelvic fin‐rays in ricefishes directly attach to the pelvic bone without in‐between radials, enlarged internal wings and basipterygial plates with medial tips could also provide more surface area for thicker rays and muscles to steer the pelvic fins. The differences between *Adrianichthys* and pelvic brooding *Oryzias* pelvic bones highlight the need for studies investigating pelvic girdle muscles and their attachment sites to elucidate if different pelvic bone shapes lead to similar effects in stabilizing the eggs while brooding. It is however also possible that the shape of the pelvic girdle is influenced not only by reproductive strategy, but also by differences between other functions in *Oryzias* and *Adrianichthys*. Studies in rainbow trout (Standen, 2008, 2010) and archerfish (Gerullis et al., 2021) show, for example, that pelvic fins and their muscles can produce precise, stabilizing movements during locomotion.

With the recent description of *O. kalimpaaensis* (Gani et al., 2022), upcoming studies likely profit from including this pelvic brooding species in their analyses, as *O. kalimpaaensis* inhabits a small lake. This contrasts the large lake habitat of species like *A. oophorus* and *O. sarasinorum* (Kottelat, 1990b; Popta, 1905), as well as the small‐pond habitat of *O. eversi* (Herder et al., 2012). Including additional ecological backgrounds in upcoming studies might provide more information on the evolution of pelvic brooding under seemingly different ecological conditions.

## 5 CONCLUSIONS

Our approach to characterize Sulawesi ricefish pelvic bones revealed that generalized Procrustes surface analysis (3D GM) can be prone to the selection of prototype shapes and the size of 3D meshes when analyzing structures showing larger morphological variation. Nevertheless, landmark‐free 3D GM successfully captured species‐ and sex‐specific differences in the medial region of complex ricefish pelvic bones and complemented 2D outline GM. By combining both approaches, we could show that females of all three examined pelvic brooding species (*Adrianichthys oophorus*, *Oryzias eversi*, *O. sarasinorum*) exhibit fine‐scaled morphological differences in their pelvic bone shape. Morphospace analyses revealed that *A. oophorus* pelvic bones resemble those of transfer brooding *Oryzias* species, which raises the question whether similar selection pressures shaped the evolution of Sulawesi ricefish pelvic girdles. Both GM approaches support the hypothesis of two independent origins of the complex pelvic brooding reproductive strategy in *Adrianichthys* and *Oryzias* ricefishes. This work lays a basis for potential future studies investigating muscular aspects of the pelvic girdle and fins, to study how hard‐ and soft‐tissues of the pelvic girdle interact in the context of pelvic brooding.

## AUTHOR CONTRIBUTIONS

**Tobias Spanke:** Conceptualization (equal); data curation (lead); formal analysis (equal); investigation (equal); methodology (equal); project administration (equal); software (equal); visualization (equal); writing – original draft (lead); writing – review and editing (lead). **Mariam Gabelaia:** Formal analysis (equal); methodology (equal); software (equal); writing – original draft (supporting); writing – review and editing (supporting). **Jana M. Flury:** Writing – original draft (supporting); writing – review and editing (supporting). **Leon Hilgers:** Writing – original draft (supporting); writing – review and editing (supporting). **Letha Louisiana Wantania:** Writing – original draft (supporting); writing – review and editing (supporting). **Bernhard Misof:** Supervision (supporting); writing – original draft (supporting); writing – review and editing (supporting). **Benjamin Wipfler:** Methodology (equal); software (equal); writing – original draft (supporting); writing – review and editing (supporting). **Daisy Wowor:** Writing – original draft (supporting); writing – review and editing (supporting). **Daniel F. Mokodongan:** Writing – original draft (supporting); writing – review and editing (supporting). **Fabian Herder:** Conceptualization (equal); funding acquisition (supporting); investigation (equal); project administration (equal); resources (lead); supervision (lead); writing – original draft (supporting); writing – review and editing (supporting). **Julia Schwarzer:** Conceptualization (equal); funding acquisition (lead); investigation (equal); project administration (equal); resources (supporting); supervision (lead); writing – original draft (supporting); writing – review and editing (supporting).

## FUNDING INFORMATION

Open Access funding enabled and organized by Projekt DEAL.

## CONFLICT OF INTEREST STATEMENT

The authors declare that they have no competing interests.

## Supporting information


Data S1.

Data S2.

Data S3.

## Data Availability

All supporting data are made freely available in the Zenodo repository (https://doi.org/10.5281/zenodo.8288625) and as Supporting Information. The μ‐CT scans are deposited at www.morphobank.org under project ID 4793.
