# Supplementary material for: A landmark‐free analysis of the pelvic girdle in Sulawesi ricefishes (Adrianichthyidae): How 2D and 3D geometric morphometrics can complement each other in the analysis of a complex structure
Source: Ecol Evol. 2023 Oct 18;13(10):e10613. doi: 10.1002/ece3.10613 (PMC10582673; doi:10.1002/ece3.10613)
Supplement: Supplementary file 1 — Data S1. [file ECE3-13-e10613-s002.docx]

**Additional Tables for: A landmark-free analysis of the pelvic girdle in Sulawesi ricefishes (Adrianichthyidae): How 2D and 3D geometric morphometrics can complement each other in the analysis of a complex structure**

T. Spanke^1^, M. Gabelaia^1^, J. Flury^1,2^, L. Hilgers^1,3^, L. L. Watania^1,4^, B. Misof^1^, B. Wipfler^1^, D. Wowor^5^, D. F. Mokodongan^5^, F. Herder^1^, J. Schwarzer^1^

^1^Leibniz Institute for the Analysis of Biodiversity Change (LIB), Museum Koenig Bonn, Adenauerallee 127, 53113 Bonn, Germany

^2^Department of Environmental Sciences, University of Basel, Basel, Switzerland

^3^LOEWE-Zentrum für Translationale Biodiversitätsgenomik, Frankfurt, Germany

^4^Faculty of Fisheries and Marine Science, Sam Ratulangi University, Manado, Indonesia

^5^Museum Zoologicum Bogoriense, Research Center for Biosystematics and Evolution, National Research and Innovation Agency (BRIN), Cibinong 16911, West Java, Indonesia

Corresponding author: Tobias Spanke

**Table S1** Collection numbers of specimens included

| **Collection number** | **Species** | **Sex** |
| --- | --- | --- |
| MZB25201 | Adrianichthys oophorus | female |
| MZB25202 | Adrianichthys oophorus | female |
| MZB25203 | Adrianichthys oophorus | female |
| MZB25204 | Adrianichthys oophorus | female |
| MZB25205 | Adrianichthys oophorus | female |
| MZB25206 | Adrianichthys oophorus | male |
| MZB25207 | Adrianichthys oophorus | male |
| MZB25208 | Adrianichthys oophorus | male |
| ZFMKICH121925 | Adrianichthys oophorus | male |
| ZFMKICH121926 | Adrianichthys oophorus | male |
| ZFMKICH121932 | Oryzias eversi | female |
| ZFMKICH121933 | Oryzias eversi | female |
| ZFMKICH121934 | Oryzias eversi | female |
| ZFMKICH121935 | Oryzias eversi | female |
| ZFMKICH121936 | Oryzias eversi | female |
| ZFMKICH121938 | Oryzias eversi | male |
| ZFMKICH121939 | Oryzias eversi | male |
| ZFMKICH121940 | Oryzias eversi | male |
| ZFMKICH121941 | Oryzias eversi | male |
| ZFMKICH121942 | Oryzias eversi | male |
| MZB25209 | Oryzias sarasinorum | female |
| MZB25210 | Oryzias sarasinorum | female |
| MZB25211 | Oryzias sarasinorum | female |
| ZFMKICH121927 | Oryzias sarasinorum | female |
| ZFMKICH121929 | Oryzias sarasinorum | female |
| MZB25212 | Oryzias sarasinorum | male |
| MZB25213 | Oryzias sarasinorum | male |
| ZFMKICH121930 | Oryzias sarasinorum | male |
| ZFMKICH121931 | Oryzias sarasinorum | male |
| ICH-126888 | Oryzias sarasinorum | male |
| MZB25214 | Oryzias celebensis | female |
| MZB25215 | Oryzias celebensis | female |
| MZB25216 | Oryzias celebensis | female |
| ZFMKICH121955 | Oryzias celebensis | female |
| ZFMKICH121956 | Oryzias celebensis | female |
| ZFMKICH121957 | Oryzias celebensis | male |
| ZFMKICH121958 | Oryzias celebensis | male |
| ZFMKICH121959 | Oryzias celebensis | male |
| ICH-126889 | Oryzias celebensis | male |
| ICH-126890 | Oryzias celebensis | male |
| M-JS19_female1 | Oryzias dopingdopingensis | female |
| ICH-126860 | Oryzias dopingdopingensis | female |
| ICH-126861 | Oryzias dopingdopingensis | female |
| ICH-126862 | Oryzias dopingdopingensis | female |
| ICH-126863 | Oryzias dopingdopingensis | female |
| M-JS19_male1 | Oryzias dopingdopingensis | male |
| ICH-126856 | Oryzias dopingdopingensis | male |
| ICH-126857 | Oryzias dopingdopingensis | male |
| ICH-126858 | Oryzias dopingdopingensis | male |
| ICH-126859 | Oryzias dopingdopingensis | male |
| ZFMKICH41828 | Oryzias matanensis | female |
| ZFMKICH41553 | Oryzias matanensis | female |
| ZFMKICH12196 | Oryzias matanensis | female |
| ZFMKICH41551 | Oryzias matanensis | female |
| ZFMKICH41831 | Oryzias matanensis | female |
| ZFMKICH41832 | Oryzias matanensis | male |
| ZFMKICH41833 | Oryzias matanensis | male |
| ZFMKICH41834 | Oryzias matanensis | male |
| ZFMKICH41835 | Oryzias matanensis | male |
| ZFMKICH41836 | Oryzias matanensis | male |
| ZFMKICH121943 | Oryzias nigrimas | female |
| ZFMKICH121944 | Oryzias nigrimas | female |
| ZFMKICH121945 | Oryzias nigrimas | female |
| ZFMKICH121947 | Oryzias nigrimas | female |
| ZFMKICH121948 | Oryzias nigrimas | female |
| ZFMKICH121950 | Oryzias nigrimas | male |
| ZFMKICH121951 | Oryzias nigrimas | male |
| ZFMKICH121952 | Oryzias nigrimas | male |
| ZFMKICH121953 | Oryzias nigrimas | male |
| ZFMKICH121954 | Oryzias nigrimas | male |
| ZFMKICH121962 | Oryzias wolasi | female |
| ZFMKICH121963 | Oryzias wolasi | female |
| ZFMKICH121964 | Oryzias wolasi | female |
| ZFMKICH121965 | Oryzias wolasi | male |
| ZFMKICH121967 | Oryzias wolasi | male |
| ZFMKICH121968 | Oryzias wolasi | male |
| ZFMKICH121966 | Oryzias wolasi | male |

**Table S2** Micro-CT scan settings

| Collection number | Species | Sex | Scanner | kV | µA | Dimensions (Px) | Pixel size (µm) | Rotation steps (Degree) | Frame Averaging | Random movement |
| --- | --- | --- | --- | --- | --- | --- | --- | --- | --- | --- |
| MZB25201 | Adrianichthys oophorus | female | Skyscan 1272 | 64 | 150 | 1344x2016 | 11 | 0.2 | 8 | 15 |
| MZB25202 | Adrianichthys oophorus | female | Skyscan 1272 | 56 | 144 | 1344x2016 | 8 | 0.2 | 8 | 15 |
| MZB25203 | Adrianichthys oophorus | female | Skyscan 1272 | 58 | 152 | 1344x2016 | 10 | 0.2 | 8 | 15 |
| MZB25204 | Adrianichthys oophorus | female | Skyscan 1272 | 60 | 150 | 1344x2016 | 8 | 0.2 | 8 | 15 |
| MZB25205 | Adrianichthys oophorus | female | Skyscan 1272 | 52 | 144 | 1344x2016 | 11 | 0.2 | 8 | 15 |
| MZB25206 | Adrianichthys oophorus | male | Skyscan 1272 | 57 | 148 | 1344x2016 | 9 | 0.2 | 8 | 15 |
| MZB25207 | Adrianichthys oophorus | male | Skyscan 1272 | 56 | 158 | 1344x2016 | 10 | 0.2 | 8 | 15 |
| MZB25208 | Adrianichthys oophorus | male | Skyscan 1272 | 56 | 144 | 1344x2016 | 8 | 0.2 | 8 | 15 |
| ZFMKICH121925 | Adrianichthys oophorus | male | Skyscan 1272 | 62144 | 140 | 1344x2016 | 10 | 0.2 | 8 | 15 |
| ZFMKICH121926 | Adrianichthys oophorus | male | Skyscan 1272 | 58 | 144 | 1344x2016 | 10 | 0.2 | 8 | 15 |
| ZFMKICH121932 | Oryzias eversi | female | Skyscan 1272 | 55 | 166 | 1344x2016 | 12 | 0.2 | 8 | 15 |
| ZFMKICH121933 | Oryzias eversi | female | Skyscan 1272 | 56 | 166 | 1344x2016 | 12 | 0.2 | 8 | 15 |
| ZFMKICH121934 | Oryzias eversi | female | Skyscan 1272 | 52 | 192 | 1344x2016 | 8 | 0.2 | 8 | 15 |
| ZFMKICH121935 | Oryzias eversi | female | Skyscan 1272 | 62 | 160 | 1344x2016 | 8 | 0.2 | 8 | 15 |
| ZFMKICH121936 | Oryzias eversi | female | Skyscan 1272 | 56 | 166 | 1344x2016 | 12 | 0.2 | 8 | 15 |
| ZFMKICH121938 | Oryzias eversi | male | Skyscan 1272 | 62 | 160 | 1344x2016 | 8 | 0.2 | 8 | 15 |
| ZFMKICH121939 | Oryzias eversi | male | Skyscan 1272 | 60 | 150 | 1344x2016 | 8 | 0.2 | 8 | 15 |
| ZFMKICH121940 | Oryzias eversi | male | Skyscan 1272 | 45 | 200 | 1344x2016 | 8 | 0.2 | 8 | 15 |
| ZFMKICH121941 | Oryzias eversi | male | Skyscan 1272 | 60 | 166 | 1344x2016 | 12 | 0.2 | 8 | 15 |
| ZFMKICH121942 | Oryzias eversi | male | Skyscan 1272 | 52 | 192 | 1344x2016 | 8 | 0.2 | 8 | 15 |
| MZB25209 | Oryzias sarasinorum | female | Skyscan 1272 | 48 | 150 | 1344x2016 | 10 | 0.2 | 8 | 15 |
| MZB25210 | Oryzias sarasinorum | female | Skyscan 1272 | 50 | 160 | 1344x2016 | 12 | 0.2 | 8 | 15 |
| MZB25211 | Oryzias sarasinorum | female | Skyscan 1272 | 56 | 150 | 1344x2016 | 12 | 0.2 | 8 | 15 |
| ZFMKICH121927 | Oryzias sarasinorum | female | Skyscan 1272 | 42 | 140 | 1344x2016 | 10 | 0.2 | 8 | 15 |
| ZFMKICH121929 | Oryzias sarasinorum | female | Skyscan 1272 | 52 | 140 | 1344x2016 | 5 | 0.2 | 8 | 15 |
| MZB25212 | Oryzias sarasinorum | male | Skyscan 1272 | 50 | 160 | 1344x2016 | 12 | 0.2 | 8 | 15 |
| MZB25213 | Oryzias sarasinorum | male | Skyscan 1272 | 44 | 140 | 1344x2016 | 9 | 0.2 | 8 | 15 |
| ZFMKICH121930 | Oryzias sarasinorum | male | Skyscan 1272 | 48 | 140 | 1344x2016 | 8 | 0.2 | 8 | 15 |
| ZFMKICH121931 | Oryzias sarasinorum | male | Skyscan 1272 | 42 | 140 | 1344x2016 | 7 | 0.2 | 8 | 15 |
| ICH-126888 | Oryzias sarasinorum | male | Skyscan 1272 | 60 | 152 | 1344x2016 | 4 | 0.2 | 8 | 15 |
| MZB25214 | Oryzias celebensis | female | Skyscan 1272 | 46 | 144 | 1344x2016 | 8 | 0.2 | 8 | 15 |
| MZB25215 | Oryzias celebensis | female | Skyscan 1272 | 44 | 144 | 1344x2016 | 8 | 0.2 | 8 | 15 |
| MZB25216 | Oryzias celebensis | female | Skyscan 1272 | 46 | 140 | 1344x2016 | 8 | 0.2 | 8 | 15 |
| ZFMKICH121955 | Oryzias celebensis | female | Skyscan 1272 | 51 | 160 | 1344x2016 | 12 | 0.2 | 8 | 15 |
| ZFMKICH121956 | Oryzias celebensis | female | Skyscan 1272 | 40 | 140 | 1344x2016 | 8 | 0.2 | 8 | 15 |
| ZFMKICH121957 | Oryzias celebensis | male | Skyscan 1272 | 52 | 160 | 1344x2016 | 12 | 0.2 | 8 | 15 |
| ZFMKICH121958 | Oryzias celebensis | male | Skyscan 1272 | 44 | 142 | 1344x2016 | 8 | 0.2 | 8 | 15 |
| ZFMKICH121959 | Oryzias celebensis | male | Skyscan 1272 | 44 | 142 | 1344x2016 | 8 | 0.2 | 8 | 15 |
| ICH-126889 | Oryzias celebensis | male | Skyscan 1272 | 44 | 142 | 1344x2016 | 8.5 | 0.2 | 8 | 15 |
| ICH-126890 | Oryzias celebensis | male | Skyscan 1272 | 56 | 144 | 1344x2016 | 8 | 0.2 | 8 | 15 |

**Table S2** Micro-CT scan settings (continued)

| Collection number | Species | Sex | Scanner | kV | µA | Dimensions (Px) | Pixel size (µm) | Rotation steps (Degree) | Frame Averaging | Random movement |
| --- | --- | --- | --- | --- | --- | --- | --- | --- | --- | --- |
| M-JS19_female1 | Oryzias dopingdopingensis | female | Skyscan 1272 | 44 | 156 | 1344x2016 | 8 | 0.2 | 8 | 15 |
| ICH-126860 | Oryzias dopingdopingensis | female | Skyscan 1272 | 44 | 140 | 1344x2016 | 7 | 0.2 | 8 | 15 |
| ICH-126861 | Oryzias dopingdopingensis | female | Skyscan 1272 | 42 | 144 | 1344x2016 | 8 | 0.2 | 8 | 15 |
| ICH-126862 | Oryzias dopingdopingensis | female | Skyscan 1272 | 42 | 140 | 1344x2016 | 8 | 0.2 | 8 | 15 |
| ICH-126863 | Oryzias dopingdopingensis | female | Skyscan 1272 | 42 | 144 | 1344x2016 | 8 | 0.2 | 8 | 15 |
| M-JS19_male1 | Oryzias dopingdopingensis | male | Skyscan 1272 | 44 | 152 | 1344x2016 | 10 | 0.2 | 8 | 15 |
| ICH-126856 | Oryzias dopingdopingensis | male | Skyscan 1272 | 44 | 142 | 1344x2016 | 7 | 0.2 | 8 | 15 |
| ICH-126857 | Oryzias dopingdopingensis | male | Skyscan 1272 | 42 | 142 | 1344x2016 | 8 | 0.2 | 8 | 15 |
| ICH-126858 | Oryzias dopingdopingensis | male | Skyscan 1272 | 44 | 144 | 1344x2016 | 7 | 0.2 | 8 | 15 |
| ICH-126859 | Oryzias dopingdopingensis | male | Skyscan 1272 | 44 | 144 | 1344x2016 | 7 | 0.2 | 8 | 15 |
| ZFMKICH41828 | Oryzias matanensis | female | Skyscan 1272 | 48 | 140 | 1344x2016 | 8 | 0.2 | 8 | 15 |
| ZFMKICH41553 | Oryzias matanensis | female | Skyscan 1272 | 50 | 160 | 1344x2016 | 12 | 0.2 | 8 | 15 |
| ZFMKICH12196 | Oryzias matanensis | female | Skyscan 1272 | 46 | 144 | 1344x2016 | 8 | 0.2 | 8 | 15 |
| ZFMKICH41551 | Oryzias matanensis | female | Skyscan 1272 | 46 | 144 | 1344x2016 | 8 | 0.2 | 8 | 15 |
| ZFMKICH41831 | Oryzias matanensis | female | Skyscan 1272 | 46 | 144 | 1344x2016 | 8 | 0.2 | 8 | 15 |
| ZFMKICH41832 | Oryzias matanensis | male | Skyscan 1272 | 48 | 144 | 1344x2016 | 8 | 0.2 | 8 | 15 |
| ZFMKICH41833 | Oryzias matanensis | male | Skyscan 1272 | 52 | 156 | 1344x2016 | 12 | 0.2 | 8 | 15 |
| ZFMKICH41834 | Oryzias matanensis | male | Skyscan 1272 | 44 | 144 | 1344x2016 | 8 | 0.2 | 8 | 15 |
| ZFMKICH41835 | Oryzias matanensis | male | Skyscan 1272 | 46 | 144 | 1344x2016 | 8 | 0.2 | 8 | 15 |
| ZFMKICH41836 | Oryzias matanensis | male | Skyscan 1272 | 46 | 144 | 1344x2016 | 8 | 0.2 | 8 | 15 |
| ZFMKICH121943 | Oryzias nigrimas | female | Skyscan 1272 | 56 | 166 | 1344x2016 | 12 | 0.2 | 8 | 15 |
| ZFMKICH121944 | Oryzias nigrimas | female | Skyscan 1272 | 56 | 166 | 1344x2016 | 12 | 0.2 | 8 | 15 |
| ZFMKICH121945 | Oryzias nigrimas | female | Skyscan 1272 | 56 | 166 | 1344x2016 | 12 | 0.2 | 8 | 15 |
| ZFMKICH121947 | Oryzias nigrimas | female | Skyscan 1272 | 62 | 160 | 1344x2016 | 8 | 0.2 | 8 | 15 |
| ZFMKICH121948 | Oryzias nigrimas | female | Skyscan 1272 | 62 | 160 | 1344x2016 | 8 | 0.2 | 8 | 15 |
| ZFMKICH121950 | Oryzias nigrimas | male | Skyscan 1272 | 60 | 166 | 1344x2016 | 8 | 0.2 | 8 | 15 |
| ZFMKICH121951 | Oryzias nigrimas | male | Skyscan 1272 | 60 | 166 | 1344x2016 | 8 | 0.2 | 8 | 15 |
| ZFMKICH121952 | Oryzias nigrimas | male | Skyscan 1272 | 50 | 160 | 1344x2016 | 12 | 0.2 | 8 | 15 |
| ZFMKICH121953 | Oryzias nigrimas | male | Skyscan 1272 | 60 | 166 | 1344x2016 | 8 | 0.2 | 8 | 15 |
| ZFMKICH121954 | Oryzias nigrimas | male | Skyscan 1272 | 60 | 166 | 1344x2016 | 8 | 0.2 | 8 | 15 |
| ZFMKICH121962 | Oryzias wolasi | female | Skyscan 1272 | 60 | 166 | 1344x2016 | 12 | 0.2 | 8 | 15 |
| ZFMKICH121963 | Oryzias wolasi | female | Skyscan 1272 | 42 | 140 | 1344x2016 | 4 | 0.2 | 8 | 15 |
| ZFMKICH121964 | Oryzias wolasi | female | Skyscan 1272 | 50 | 148 | 1344x2016 | 7 | 0.2 | 8 | 15 |
| ZFMKICH121965 | Oryzias wolasi | male | Skyscan 1272 | 54 | 160 | 1344x2016 | 12 | 0.2 | 8 | 15 |
| ZFMKICH121967 | Oryzias wolasi | male | Skyscan 1272 | 48 | 160 | 1344x2016 | 8 | 0.2 | 8 | 15 |
| ZFMKICH121968 | Oryzias wolasi | male | Skyscan 1272 | 44 | 140 | 1344x2016 | 8 | 0.2 | 8 | 15 |
| ZFMKICH121966 | Oryzias wolasi | male | Skyscan 1272 | 42 | 144 | 1344x2016 | 8 | 0.2 | 8 | 15 |

**Table S3** Individual and cumulative loadings for PC axes 1-10 in 2D and 3D shape analysis.

|  | **2D outline analysis PC loading** | | **3D surface analysis PC loading** | |
| --- | --- | --- | --- | --- |
| PC axis | Individual (%) | Cumulative (%) | Individual (%) | Cumulative (%) |
| 1 | 52.2634 | 52.2634 | 18 | 18 |
| 2 | 10.4774 | 62.7408 | 11.2 | 29.2 |
| 3 | 8.1211 | 70.8619 | 8.82 | 38.02 |
| 4 | 5.3569 | 76.2188 | 4.99 | 43.01 |
| 5 | 4.5889 | 80.8077 | 4.31 | 47.32 |
| 6 | 3.5606 | 84.3683 | 3.7 | 51.03 |
| 7 | 2.8956 | 87.2639 | 3.32 | 54.35 |
| 8 | 2.2226 | 89.4865 | 2.96 | 57.3 |
| 9 | 1.8398 | 91.3263 | 2.34 | 59.64 |
| 10 | 1.1997 | 93.3233 | 2.12 | 61.76 |

**Table S4 PERMANOVA model for 2D outline and 3D GPSA analysis.** Summary of the PERMANOVA analysis performed with the 2D outline and 3D GPSA datasets (PC1 to PC6) using the ‘vegan’ R package. Selected PC axes were estimated with the broken stick method. Interaction of effects ‘reproductive strategy’ and ‘sex’ marked with asterisk. Significance levels of p < 0.05 are highlighted in bold.

| **2D outline dataset** |  |  |  |  |  |  |
| --- | --- | --- | --- | --- | --- | --- |
| **Fixed effect** | *Permutations* | *num DF* | *Sum of Squares* | *R2* | *F* | *P* |
| Reproductive strategy | 9999 | 1 | 0.449 | 0.233 | 48.37 | **0.0001** |
| Sex | 9999 | 1 | 0.022 | 0.011 | 2.38 | 0.07 |
| Species | 9999 | 6 | 0.766 | 0.4 | 13.78 | **0.0001** |
| Rep-Strategy*Sex | 9999 | 1 | 0.078 | 0.041 | 8.42 | **0.0003** |
| **Covariate** |  |  |  |  |  |  |
| Standard length | 9999 | 1 | 0.019 | 0.041 | 8.55 | 0.11 |
| **3D GPSA dataset** |  |  |  |  |  |  |
| **Fixed effect** | *Permutations* | *num DF* | *Sum of Squares* | R2 | *F* | *P* |
| Reproductive strategy | 9999 | 1 | 1122.7 | 0.116 | 12.91 | **0.0001** |
| Sex | 9999 | 1 | 115.5 | 0.012 | 1.33 | 0.24 |
| Species | 9999 | 6 | 2605 | 0.269 | 4.99 | **0.0001** |
| Rep-Strategy*Sex | 9999 | 1 | 269.6 | 0.028 | 3.1 | **0.01** |
| **Covariate** |  |  |  |  |  |  |
| Standard length | 9999 | 1 | 49.8 | 0.005 | 0.57 | 0.72 |

**Table S5 GPSA specimen alignment.** Face number and prototype combinations were tested five times to test for possible variations when aligning pelvic girdle shapes. The first run of each combination that aligned the maximum amount (74) of pelvic girdles was selected for the disparity analysis to select the best-fitting combination for the GPSA shape analysis. Row coloration indicates the two different prototypes.

| **GPSA dataset** | **Prototype** | **Failed alignment** | **Qualitative description (each individual respectively)** | **# Aligned** | **# Misaligned** |
| --- | --- | --- | --- | --- | --- |
| 200,000 run 1 | MZB25203 | ICH-126862, ZFMKICH121935, ZFMKICH121936 | Mirrored alignment, 180° rotated, slightly rotated | 73 | 3 |
| 200,000 run 2 | MZB25203 | ICH-126862, ZFMKICH121935, ZFMKICH121936 | Mirrored alignment, 180° rotated, 180° rotated | 73 | 3 |
| 200,000 run 3 | MZB25203 | ICH-126862, ZFMKICH121935 | Mirrored alignment, 180° rotated | 74 | 2 |
| 200,000 run 4 | MZB25203 | ICH-126862, ZFMKICH121935, ZFMKICH121936 | Mirrored alignment, 180° rotated, slightly rotated | 73 | 3 |
| 200,000 run 5 | MZB25203 | ICH-126862, ZFMKICH121935 | Mirrored alignment, 180° rotated | 74 | 2 |
| 200,000 run 1 | MZB25205 | ICH-126862, ZFMKICH121935, ZFMKICH121936 | Mirrored alignment, 180° rotated, 180° rotated | 73 | 3 |
| 200,000 run 2 | MZB25205 | ICH-126862, ZFMKICH121935, ZFMKICH121936 | Mirrored alignment, 180° rotated, slightly rotated | 73 | 3 |
| 200,000 run 3 | MZB25205 | ICH-126862, ZFMKICH121935, ZFMKICH121936 | Mirrored alignment, 180° rotated, 180° rotated | 73 | 3 |
| 200,000 run 4 | MZB25205 | ICH-126862, ZFMKICH121935, ZFMKICH121936 | Mirrored alignment, 180° rotated, slightly rotated | 73 | 3 |
| 200,000 run 5 | MZB25205 | ICH-126862, ZFMKICH121935, ZFMKICH121936 | Mirrored alignment, 180° rotated, slightly rotated | 73 | 3 |
| 500,000 run 1 | MZB25203 | ICH-126862, ZFMKICH121935 | Mirrored alignment, 180° rotated | 74 | 2 |
| 500,000 run 2 | MZB25203 | ICH-126862, ZFMKICH121935, ZFMKICH121936 | Mirrored alignment, 180° rotated, slightly rotated | 73 | 3 |
| 500,000 run 3 | MZB25203 | ICH-126862, ZFMKICH121935, ZFMKICH121936 | Mirrored alignment, 180° rotated, 180° rotated | 73 | 3 |
| 500,000 run 4 | MZB25203 | ICH-126862, ZFMKICH121935, ZFMKICH121936 | Mirrored alignment, 180° rotated, 180° rotated | 73 | 3 |
| 500,000 run 5 | MZB25203 | ICH-126862, ZFMKICH121935 | Mirrored alignment, 180° rotated | 74 | 2 |
| 500,000 run 1 | MZB25205 | ICH-126862, ZFMKICH121935, ZFMKICH121936 | Mirrored alignment, 180° rotated, slightly rotated | 73 | 3 |
| 500,000 run 2 | MZB25205 | ICH-126862, ZFMKICH121935 | Mirrored alignment, 180° rotated | 74 | 2 |
| 500,000 run 3 | MZB25205 | ICH-126862, ZFMKICH121935 | Mirrored alignment, 180° rotated | 74 | 2 |
| 500,000 run 4 | MZB25205 | ICH-126862, ZFMKICH121933, ZFMKICH121935 | Mirrored alignment, slightly rotated, 180° rotated | 73 | 3 |
| 500,000 run 5 | MZB25205 | ICH-126862, ZFMKICH121935, ZFMKICH121936 | Mirrored alignment, 180° rotated, 180° rotated | 73 | 3 |
| 800,000 run 1 | MZB25203 | ICH-126857, ICH-126862, ZFMKICH121935 | Mirrored alignment, mirrored alignment, 180° rotated | 73 | 3 |
| 800,000 run 2 | MZB25203 | ICH-126857, ICH-126862, ZFMKICH121935 | Mirrored alignment, mirrored alignment, Mirrored alignment+180° rotated | 73 | 3 |
| 800,000 run 3 | MZB25203 | ICH-126862, ZFMKICH121935, ZFMKICH121936 | Mirrored alignment, 180° rotated, offset alignment | 73 | 3 |
| 800,000 run 4 | MZB25203 | ICH-126862, ZFMKICH121935 | Mirrored alignment, mirrored alignment+180° rotated | 74 | 2 |
| 800,000 run 5 | MZB25203 | ICH-126862, ZFMKICH121935 | Mirrored alignment, 180° rotated | 74 | 2 |
| 800,000 run 1 | MZB25205 | ICH-126862, ZFMKICH121935, ZFMKICH121967 | Mirrored alignment, mirrored alignment+180° rotated, mirrored alignment | 73 | 3 |
| 800,000 run 2 | MZB25205 | ICH-126862, ZFMKICH121935, ZFMKICH121967 | Mirrored alignment, mirrored alignment+180° rotated, mirrored alignment | 73 | 3 |
| 800,000 run 3 | MZB25205 | ICH-126862, ZFMKICH121935, ZFMKICH121936, ZFMKICH121967 | Mirrored, mirrored+180° rotated, mirrored+180° rotated, mirrored | 72 | 4 |
| 800,000 run 4 | MZB25205 | ICH-126862, ZFMKICH121935, ZFMKICH121936, ZFMKICH121967 | Mirrored, mirrored+180° rotated, mirrored+180° rotated, mirrored | 72 | 4 |
| 800,000 run 5 | MZB25205 | ICH-126862, ZFMKICH121935, ZFMKICH121936, ZFMKICH121967 | Mirrored, mirrored+180° rotated, mirrored+180° rotated, mirrored | 72 | 4 |

**Table S6 Disparity metrics**. Disparity scores of the ‘convex hull surface’ and ‘range’ metric calculated using the ‘dispRity’ R package (R 4.1.1). Dataset and prototype combinations were tested to identify the best-fitting combination used in the final GPSA shape analysis. Differing letters in the column ’Dataset differences’ indicate significant differences between datasets and their corresponding disparity score. Detailed pairwise comparisons are given in Table S7.

| **Convex hull surface** | |  |  |  |  |  |  |  |  |
| --- | --- | --- | --- | --- | --- | --- | --- | --- | --- |
| **Mesh face size** | **Prototype** | **N** | **Disparity score** | **Bootstrap score** | **Dataset differences (t.statistics)** | **2.5% interval** | **25% interval** | **75% interval** | **97.5% interval** |
| 200,000 | MZB25203 | 74 | 877.58 | 454.72 | a | 213.13 | 356.29 | 587.46 | 738 |
| 500,000 | MZB25203 | 74 | 937.52 | 485.92 | b | 219.69 | 381.15 | 596.45 | 777.04 |
| 500,000 | MZB25205 | 74 | 1126.48 | 508.09 | c | 216.9 | 372.03 | 685.63 | 935.18 |
| 800,000 | MZB25203 | 74 | 882.6 | 485.88 | b | 245.32 | 399.27 | 575.75 | 713.58 |
| **PC score ranges** | |  |  |  |  |  |  |  |  |
| **Mesh face size** | **Prototype** | **N** | **Disparity score** | **Bootstrap score** | **Dataset differences (t.statistics)** | **2.5% interval** | **25% interval** | **75% interval** | **97.5% interval** |
| 200,000 | MZB25203 | 74 | 34.98 | 30.8966 | a | 25.107 | 29.454 | 33.7591 | 34.8088 |
| 500,000 | MZB25203 | 74 | 33.5 | 30.1387 | b | 25.434 | 28.524 | 31.6934 | 33.3936 |
| 500,000 | MZB25205 | 74 | 35.42 | 31.5216 | c | 25.916 | 29.592 | 33.253 | 35.2407 |
| 800,000 | MZB25203 | 74 | 31.1 | 28.9798 | d | 25.45 | 27.684 | 30.0048 | 30.9563 |

**Table S7 Disparity statistics.** Pairwise comparisons of dataset face numbers and prototype combinations for the ‘convex hull surface’ and ‘range’ metric calculated using the ‘dispRity’ R package (R 4.1.1). Dataset and prototype combinations were used to identify the best-fitting combination for GPSA shape analysis. The comparison was performed using the test.dispRity function of the ‘dispRity’ R package. Significance levels p < 0.05 are highlighted in bold.

| **Convex hull surface** |  |  |  |  |  |
| --- | --- | --- | --- | --- | --- |
| **Pairwise comparison** |  | **T statistics** | **DF** | **P value** | **Std. error** |
| 200,000:MZB25203 - 500,000:MZB25203 | | -7.411791 | 9997.286 | **0.0000** | 2.91725 |
| 200,000:MZB25203 - 500,000:MZB25205 | | -18.189745 | 9131.825 | **0.0000** | 3.491979 |
| 200,000:MZB25203 - 800,000:MZB25203 | | -6.350367 | 9720.99 | **0.0000** | 2.686947 |
| 500,000:MZB25203 - 500,000:MZB25205 | | -11.962596 | 9170.906 | **0.0000** | 3.502263 |
| 500,000:MZB25203 - 800,000:MZB25203 | | 1.688314 | 9694.264 | 0.5483 | 2.700298 |
| 500,000:MZB25205 - 800,000:MZB25203 | | 14.022572 | 8294.248 | **0.0000** | 3.312881 |
| **PC score ranges** |  |  |  |  |  |
| **Pairwise comparison** |  | **T statistics** | **DF** | **P value** | **Std. error** |
| 200,000:MZB25203 - 500,000:MZB25203 | | 19.973663 | 9311.227 | **0.000000** | 0.05032221 |
| 200,000:MZB25203 - 500,000:MZB25205 | | -4.876439 | 9854.624 | **0.000007** | 0.05360471 |
| 200,000:MZB25203 - 800,000:MZB25203 | | 49.546474 | 7692.11 | **0.000000** | 0.04561572 |
| 500,000:MZB25203 - 500,000:MZB25205 | | -27.09029 | 9760.242 | **0.000000** | 0.04675177 |
| 500,000:MZB25203 - 800,000:MZB25203 | | 33.624217 | 9047.476 | **0.000000** | 0.03732368 |
| 500,000:MZB25205 - 800,000:MZB25203 | | 60.54934 | 8270.164 | **0.000000** | 0.0416437 |
