# Supplementary material for: A landmark‐free analysis of the pelvic girdle in Sulawesi ricefishes (Adrianichthyidae): How 2D and 3D geometric morphometrics can complement each other in the analysis of a complex structure
Source: Ecol Evol. 2023 Oct 18;13(10):e10613. doi: 10.1002/ece3.10613 (PMC10582673; doi:10.1002/ece3.10613)
Supplement: Supplementary file 2 — Data S2. [file ECE3-13-e10613-s003.docx]

**Additional Figures for: A landmark-free analysis of the pelvic girdle in Sulawesi ricefishes (Adrianichthyidae): How 2D and 3D geometric morphometrics can complement each other in the analysis of a complex structure**

T. Spanke^1^, M. Gabelaia^1^, J. Flury^1,2^, L. Hilgers^1,3^, L. L. Watania^1,4^, B. Misof^1^, B. Wipfler^1^, D. Wowor^5^, D. F. Mokodongan^5^, F. Herder^1^, J. Schwarzer^1^

^1^Leibniz Institute for the Analysis of Biodiversity Change (LIB), Museum Koenig Bonn, Adenauerallee 127, 53113 Bonn, Germany

^2^Department of Environmental Sciences, University of Basel, Basel, Switzerland

^3^LOEWE-Zentrum für Translationale Biodiversitätsgenomik, Frankfurt, Germany

^4^Faculty of Fisheries and Marine Science, Sam Ratulangi University, Manado, Indonesia

^5^Museum Zoologicum Bogoriense, Research Center for Biosystematics and Evolution, National Research and Innovation Agency (BRIN), Cibinong 16911, West Java, Indonesia

Corresponding author: Tobias Spanke


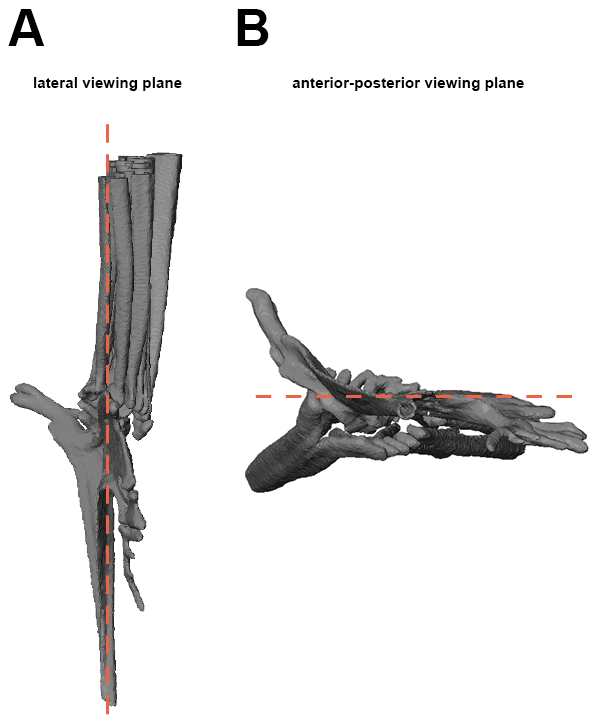


**Fig. S1:** Illustration showing the orientation of an exemplary right pelvic bone for the 2D outline shape analysis. Surface renders were rotated so that in lateral viewing plane the basipterygial arm was aligned vertically (A, dashed line). Simultaneously, in anterior-posterior orientation the medial part of the pelvic bone was oriented horizontally (B, dashed line).


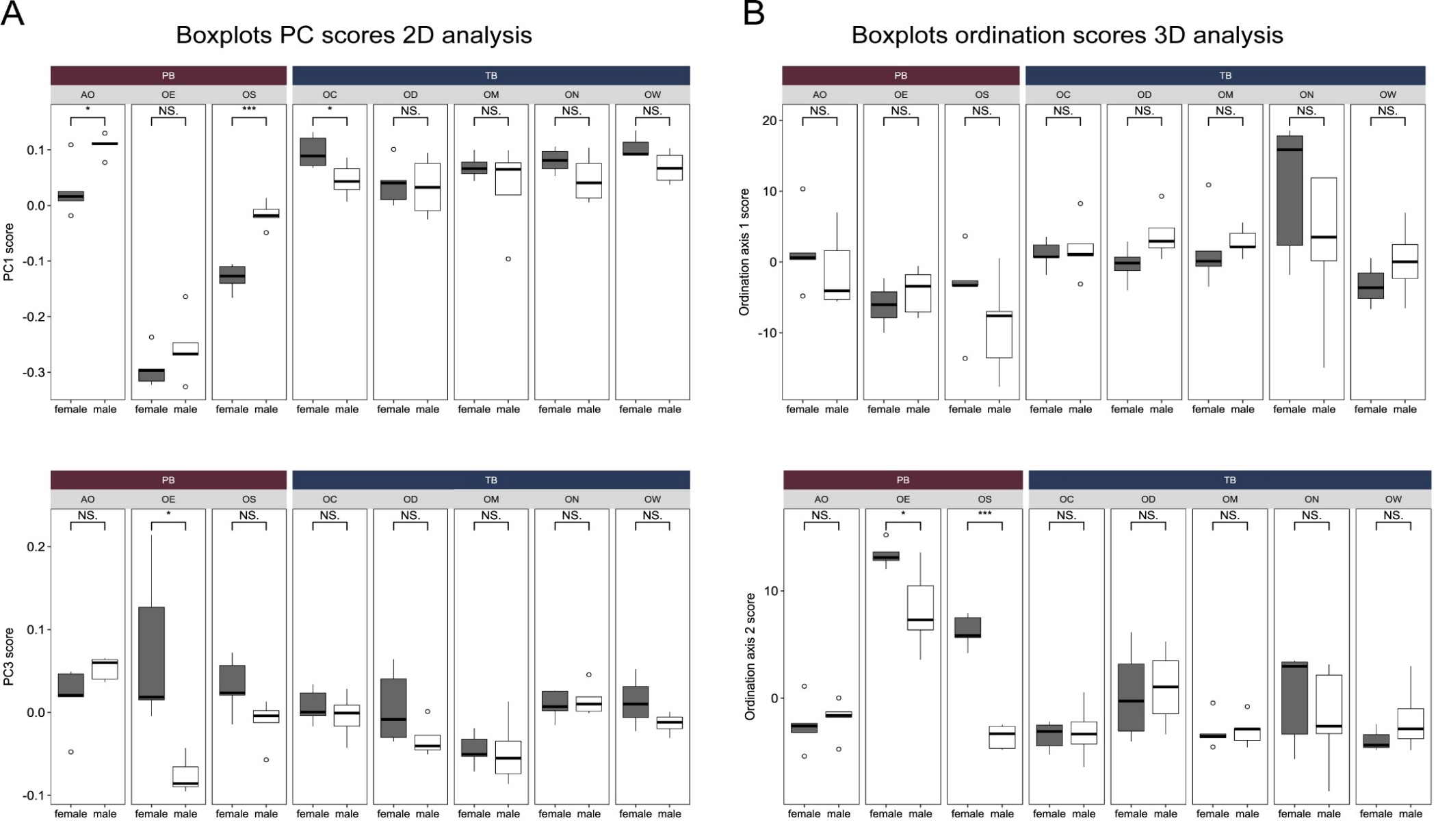
**Fig. S2:** Boxplots showing PC (2D analysis) and ordination scores (3D analysis) separated for female (dark) and male (light) specimens. Sexual dimorphism is more prominent in pelvic brooding species (T-test statistics). Coloration indicates pelvic (red) and transfer (blue) brooding species. AO = *A. oophorus*; OE = *O. eversi*; OS = *O. sarasinorum*; OC = *O. celebensis*; OD = *O. dopingdopingensis*; OM = *O. matanensis*; ON = *O. nigrimas*; OW = *O. wolasi*.

**
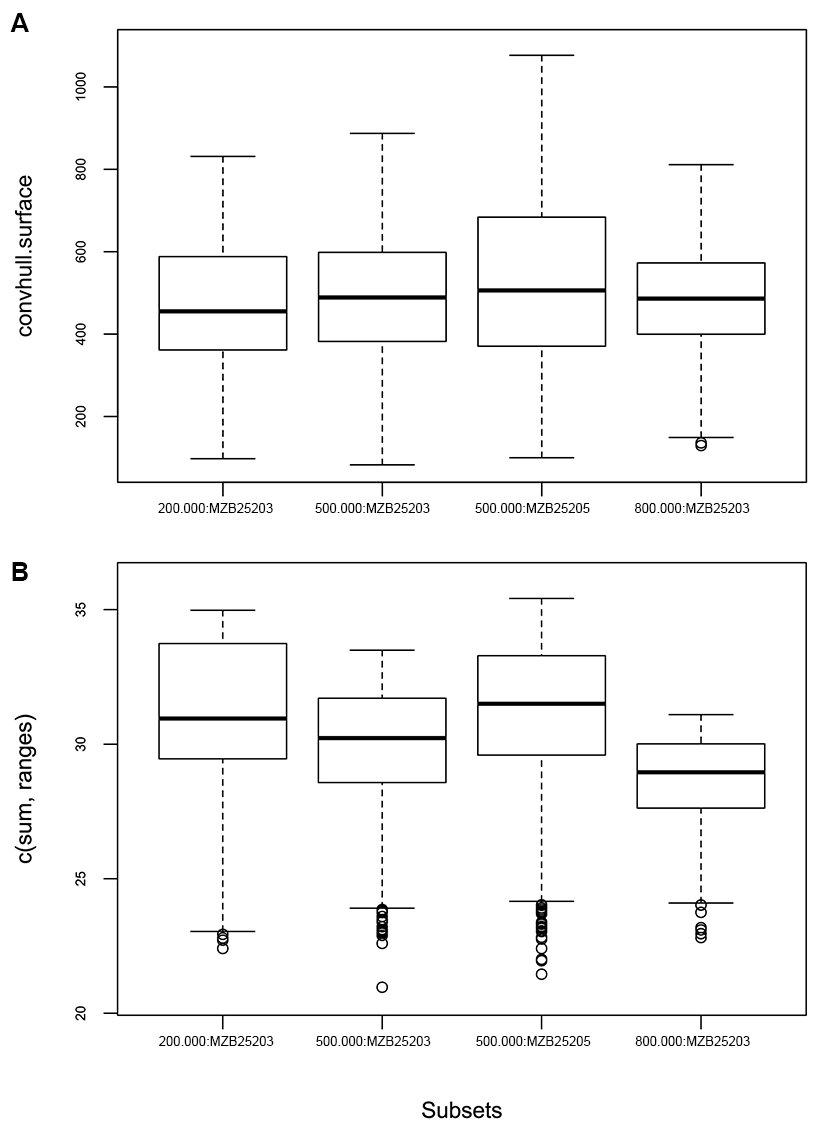
Fig. S3:** Boxplots showing disparity scores estimated for mesh size (200,000, 500,000, 800,000) and prototype (MZB25203, MZB25205) combinations. Whiskers indicate 25% to 75% quartiles, with median in bold. A: Convex hull surface was smallest in the 200,000:MZB25203 combination. B: Prototype MZB25203 showed the highest ranges when paired with the 200.000 face dataset. The overall highest ranges were estimated in the 500,000:MZB25205 combination.


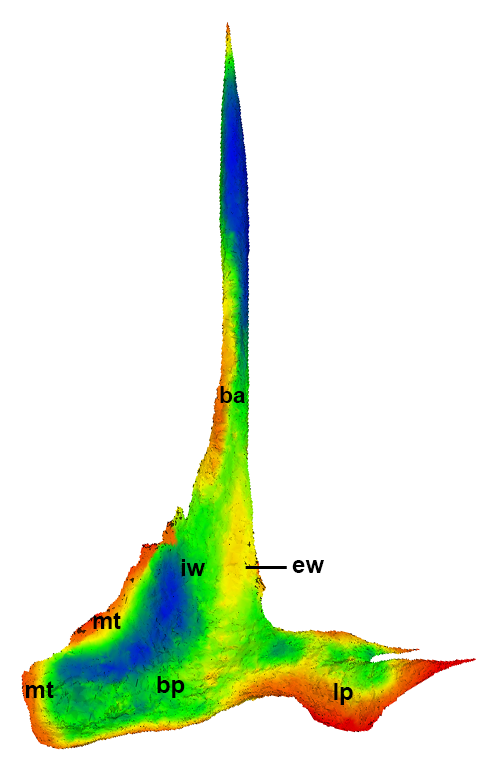


**Fig. S4:** Mean shape of the right pelvic bone calculated by GPSA with overlayed heatmap coding for shape variation for all PC axes of the 76 analyzed pelvic bones. Coding ranges from blue to red representing small to high degrees of morphological differences, respectively. High variation (red colors) is found in the lateral process within the dataset. Additionally, the regions on the medial edges of the pelvic bone where medial tips (mt) are located in pelvic brooding Oryzias show red coloration and point towards the strong differences seen between pelvic brooding species (O. eversi, O. sarasinorum) and remaining ricefishes. Moreover, the transition zone of the basipterygial arm (ba) to the internal wing is marked in orange, indicating additional variation among pelvic bones in this region.


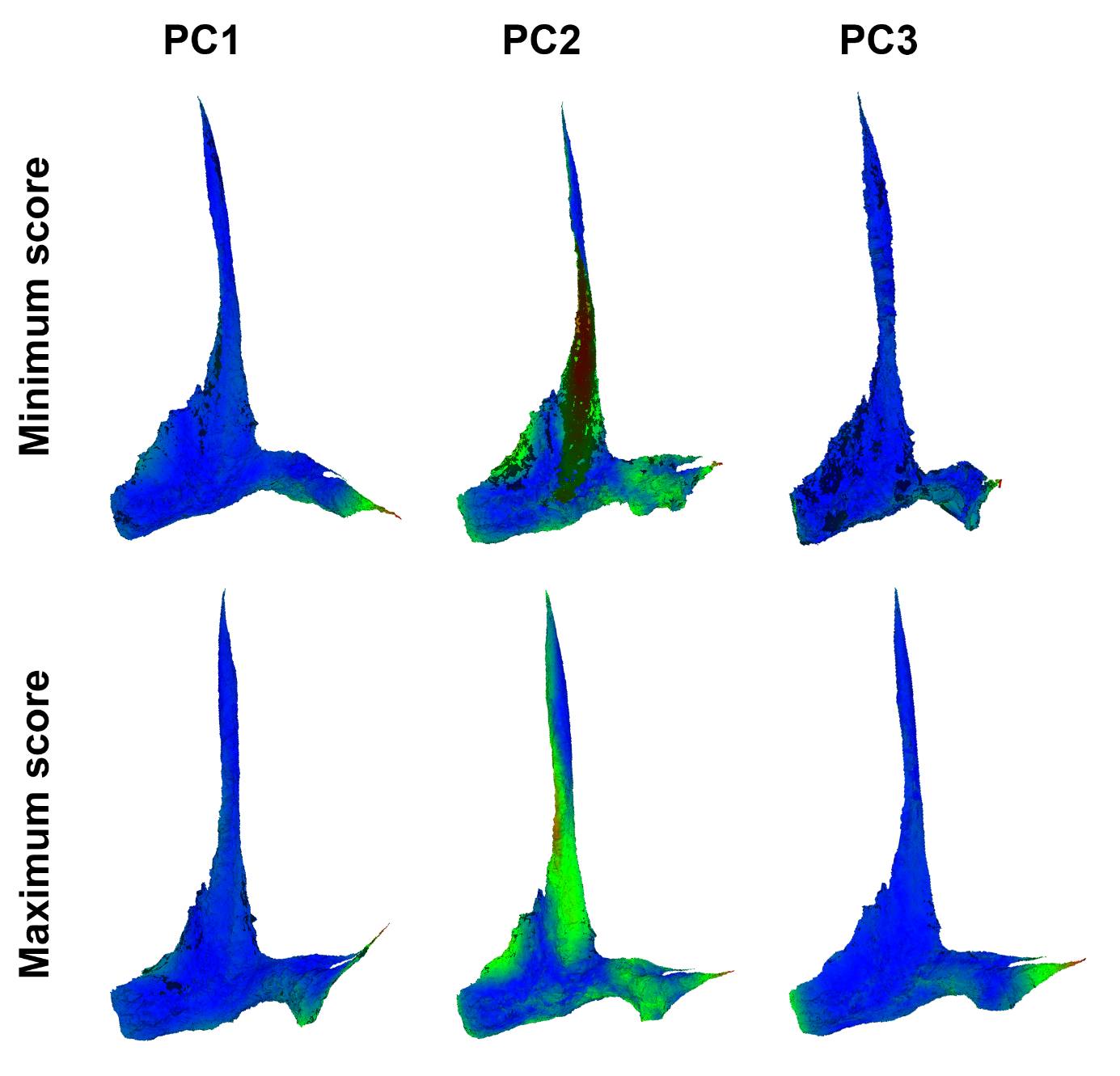


**Fig. S5:** Two-dimensional representations of right pelvic bone mean shapes of the 200,000:WF8 (3D landmark-free) dataset with overlayed heatmaps depicting shape changes over PC axes 1-3. Coloration indicates areas with low (blue) to stronger (green to red) variations within the dataset.


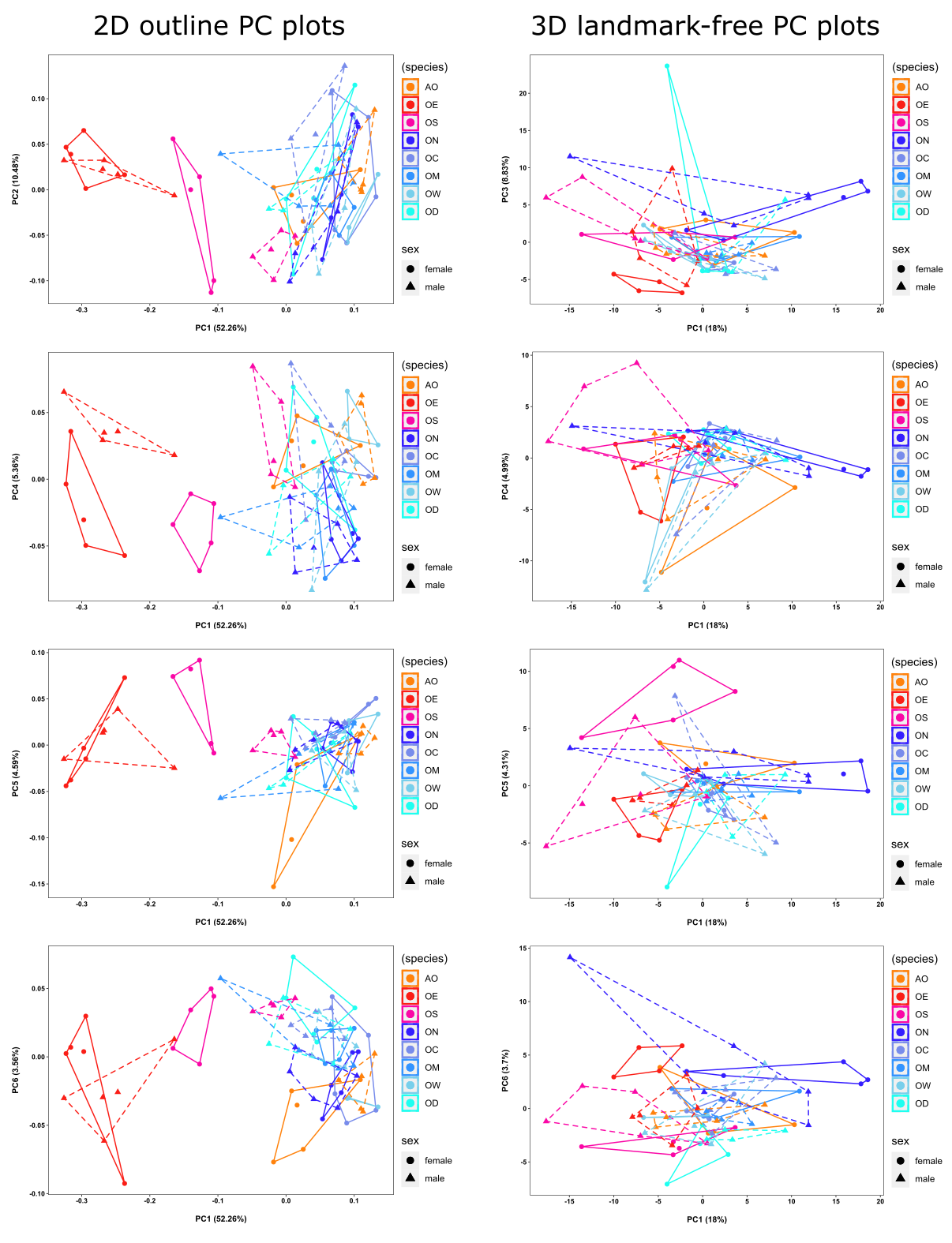


**Fig. S6:** Plate showing additional PC plots of the 2D outline (left) and 3D landmark-free (right) analyses not depicted in the main manuscript.
